# Supplementary material for: Diverse values of nature for sustainability
Source: Nature. 2023 Aug 9;620(7975):813–23. doi: 10.1038/s41586-023-06406-9 (PMC10447232; doi:10.1038/s41586-023-06406-9)
Supplement: Supplementary file 1 — The Supplementary Information includes three parts. Part A explains how the paper is associated with the IPBES Values Assessment. Part B provides details about each of the 29 review protocols. Part C offers information about the case study of Chilika Lagoon, India, that is used in the main paper. [file 41586_2023_6406_MOESM1_ESM.docx]

**SUPPLEMENTARY INFORMATION**

**Diverse values of nature for sustainability**

Unai Pascual^1,2,3^ ✉, Patricia Balvanera^4^, Christopher B. Anderson^5,6^, Rebecca Chaplin-Kramer^7^,^8^, Michael Christie^9^, David González-Jiménez^4,10^, Adrian Martin^11^, Christopher M. Raymond^12,13,14^, Mette Termansen^15^, Arild Vatn^16^, Simone Athayde^17^, Brigitte Baptiste^18^, David N. Barton^19^, Sander Jacobs^20,21^, Eszter Kelemen^22^, Ritesh Kumar^23^, Elena Lazos^24^, Tuyeni H. Mwampamba^4,25^, Barbara Nakangu^26^, Patrick O’Farrell^27,28^, Suneetha M. Subramanian^29^, Meine van Noordwijk^30,31,32^, SoEun Ahn^33^, Sacha Amaruzaman^30^, Ariane M. Amin^34,35^, Paola Arias-Arévalo^36^, Gabriela Arroyo-Robles^4^, Mariana Cantú-Fernández^4^, Antonio J. Castro^37^, Victoria Contreras^4^, Alta De Vos^38,39^, Nicolas Dendoncker^40^, Stefanie Engel^41^, Uta Eser^42^, Daniel P. Faith^43^, Anna Filyushkina^44,45^, Houda Ghazi^46^, Erik Gómez-Baggethun^16,19^, Rachelle K. Gould^47^, Louise Guibrunet^48^, Haripriya Gundimeda^49^, Thomas Hahn^50^, Zuzana V. Harmáčková^50,51^, Marcello Hernández-Blanco^52^, Andra-Ioana Horcea-Milcu^53,54^, Mariaelena Huambachano^55^, Natalia Lutti Hummel Wicher^56^, Cem İskender Aydın^57^, Mine Islar^58^, Ann-Kathrin Koessler^40,59^, Jasper O. Kenter^9,60,61^, Marina Kosmus^62^, Heera Lee^63,64^, Beria Leimona^30^, Sharachchandra Lele^65,66,67^, Dominic Lenzi^68^, Bosco Lliso^1,69^, Lelani M. Mannetti^70^, Juliana Merçon^71^, Ana Sofía Monroy-Sais^72^, Nibedita Mukherjee^73^, Barbara Muraca^74^, Roldan Muradian^75^, Ranjini Murali^76,77^, Sara H. Nelson^78^, Gabriel R. Nemogá-Soto^79,80^, Jonas Ngouhouo-Poufoun^81,82^, Aidin Niamir^83^, Emmanuel Nuesiri^84^, Tobias O. Nyumba^61,85^, Begüm Özkaynak^86^, Ignacio Palomo^87^, Ram Pandit^88,89^, Agnieszka Pawłowska-Mainville^90,91^, Luciana Porter-Bolland^92^, Martin Quaas^93^, Julian Rode^94^, Ricardo Rozzi^95,96^, Sonya Sachdeva^97^, Aibek Samakov^98^, Marije Schaafsma^44,99^, Nadia Sitas^39^, Paula Ungar^100^, Evonne Yiu^101^, Yuki Yoshida^102^ & Eglee Zent^103^

^1^Basque Centre for Climate Change (BC3), Scientific Campus of the University of the Basque Country, Leioa, Spain. ^2^Ikerbasque Basque Foundation for Science, Bilbao, Spain. ^3^Centre for Development and Environment, University of Bern, Bern, Switzerland. ^4^Instituto de Investigaciones en Ecosistemas y Sustentabilidad, Universidad Nacional Autónoma de México (UNAM), Morelia, México. ^5^Instituto de Ciencias Polares, Ambiente y Recursos Naturales, Universidad Nacional de Tierra del Fuego (ICPA-UNTDF), Ushuaia, Argentina. ^6^Centro Austral de Investigaciones Científicas, Consejo Nacional de Investigaciones Científicas y Técnicas (CADIC-CONICET), Ushuaia, Argentina. ^7^Global Science, WWF, San Francisco, CA, USA. ^8^Institute on the Environment, University of Minnesota, St. Paul, MN, USA. ^9^Aberystwyth Business School, Aberystwyth University, Aberystwyth, UK. ^10^Global Resilience Partnership, Cape Town, South Africa. ^11^School of International Development, University of East Anglia, Norwich, UK. ^12^Helsinki Institute of Sustainability Science, University of Helsinki, Helsinki, Finland. ^13^Ecosystems and Environment Research Program, Faculty of Biological and Environmental Sciences, University of Helsinki, Helsinki, Finland. ^14^Department of Economics and Management, University of Helsinki, Helsinki, Finland. ^15^Department of Food and Resource Economics, University of Copenhagen, Copenhagen, Denmark. ^16^Department of International Environment and Development Studies, Norwegian University of Life Sciences, Ås, Norway. ^17^Department of Global and Sociocultural Studies and Kimberly Green Latin American and Caribbean Center, Florida International University, Miami, FL, USA. ^18^University of EAN, Bogotá, Colombia. ^19^Norwegian Institute for Nature Research (NINA), Oslo, Norway. ^20^Research Institute for Nature and Forest INBO, Brussels, Belgium. ^21^Belgian Biodiversity Platform, Brussels, Belgium. ^22^ESSRG Nonprofit Kft., Budapest, Hungary. ^23^Wetlands International South Asia, New Delhi, India. ^24^Instituto de Investigaciones Sociales, Universidad Nacional Autónoma de México (UNAM), Mexico City, México. ^25^Department of Ecosystems and Conservation, College of Forestry, Wildlife and Tourism, Sokoine University of Agriculture, Morogoro, Tanzania. ^26^World Wide Fund for Nature (WWF), Culemborg, The Netherlands. ^27^Department of Biodiversity and Conservation Biology, Faculty of Natural Sciences, University of the Western Cape, Cape Town, South Africa. ^28^Institute for Integrated Management of Material Fluxes and of Resources, UNU-FLORES, United Nations University, Dresden, Germany. ^29^Institute for the Advanced Study of Sustainability, United Nations University, Tokyo, Japan. ^30^International Centre for Forestry Research and World Agroforestry (CIFOR-ICRAF), Bogor, Indonesia. ^31^Plant Production Systems, Wageningen University and Research, Wageningen, The Netherlands. ^32^Agroforestry Research Group, Brawijaya University, Malang, Indonesia. ^33^Korea Environment Institute, Sejong, Republic of Korea. ^34^Université Felix Houphouët-Boigny, Abidjan, Côte d’Ivoire. ^35^Centre Suisse de Recherche Scientifique, Abidjan, Côte d’Ivoire. ^36^Departamento de Economía, Facultad de Ciencias Sociales y Económicas, Universidad del Valle, Cali, Colombia. ^37^Departamento de Biología y Geología, Centro Andaluz de Evaluación y Seguimiento del Cambio Global (CAESCG), Universidad de Almería, Almería, Spain. ^38^Department of Environmental Science, Rhodes University, Grahamstown, South Africa. ^39^Centre for Sustainability Transitions, Stellenbosch University, Stellenbosch, South Africa. ^40^Department of Geography, Institute of Life Earth and Environment, University of Namur, Namur, Belgium. ^41^School of Business Administration and Economics & Institute for Environmental Systems Research, Osnabrück University, Osnabrück, Germany. ^42^Office for Environmental Ethics, Tübingen, Germany. ^43^Charles Perkins Centre, The University of Sydney, Sydney, New South Wales, Australia. ^44^Department of Ecology, Swedish University of Agricultural Sciences, Uppsala, Sweden. ^45^Institute for Environmental Studies, Vrije University Amsterdam, Amsterdam, The Netherlands. ^46^OCP Foundation, Casablanca, Morocco. ^47^Rubenstein School of Environment and Natural Resources, University of Vermont, Burlington, VT, USA. ^48^Institute of Geography, National Autonomous University of México (UNAM), Mexico City, México. ^49^Department of Economics, Indian Institute of Technology Bombay, Mumbai, India. ^50^Stockholm Resilience Centre, Stockholm University, Stockholm, Sweden. ^51^Global Change Research Institute of the Czech Academy of Sciences, Brno, Czech Republic. ^52^Independent scholar, San José, Costa Rica. ^53^Kassel Institute for Sustainability, University of Kassel, Kassel, Germany. ^54^Faculty of Humanities and Cultural Studies, University of Kassel, Kassel, Germany. ^55^Center for Global Indigenous Cultures and Environmental Justice Center, Syracuse University, New York, NY, USA. ^56^Escola de Administração de Empresas de São Paulo da Fundação Getúlio Vargas, São Paulo, Brazil. ^57^Institute of Environmental Sciences, Boğaziçi University, Istanbul, Turkey. ^58^Center for Sustainability Studies, Lund University, Lund, Sweden. ^59^Institute of Environmental Planning, Leibniz University Hannover, Hannover, Germany. ^60^Ecologos Research Ltd, Aberystwyth, UK. ^61^Department of Environment and Geography, University of York, York, UK. ^62^Deutsche Gesellschaft für Internationale Zusammenarbeit GIZ, Bonn, Germany. ^63^Department of Forestry and Landscape Architecture, Konkuk University, Seoul, Republic of Korea. ^64^Karlsruhe Institute of Technology (KIT), Institute of Meteorology and Climate Research, Atmospheric Environmental Research (IMK-IFU), Garmisch-Partenkirchen, Germany. ^65^Centre for Environment & Development, ATREE, Bengaluru, India. ^66^Indian Institute of Science Education & Research, Pune, India. ^67^Shiv Nadar University, Delhi, India. ^68^Department of Philosophy, University of Twente, Enschede, The Netherlands. ^69^World Benchmarking Alliance, Amsterdam, The Netherlands. ^70^Urban Studies Institute, Georgia State University, Atlanta, GA, USA. ^71^Instituto de Investigaciones en Educación, Universidad Veracruzana, Xalapa, México. ^72^Centro de Investigaciones en Geografía Ambiental, Universidad Nacional Autónoma de México (UNAM), Morelia, México. ^73^Division of Anthropology, Geography and Development, Department of Social and Political Sciences, Brunel University, London, UK. ^74^Department of Philosophy and Environmental Studies Program, University of Oregon, Eugene, OR, USA. ^75^Faculdade de Economia, Universidade Federal Fluminense, Niterói, Brazil. ^76^The Snow Leopard Trust, Seattle, WA, USA. ^77^Geography Department, Humboldt Universität zu Berlin, Berlin, Germany. ^78^Centre for Climate Justice, University of British Columbia, Vancouver, British Columbia, Canada. ^79^University of Winnipeg, Winnipeg, Manitoba, Canada. ^80^Universidad Nacional de Colombia, Bogotá, Colombia. ^81^International Institute of Tropical Agriculture (IITA), Nkolbisson Yaoundé, Cameroon. ^82^Congo Basin Institute (CBI), Nkolbisson Yaoundé, Cameroon. ^83^Senckenberg Biodiversity and Climate Research Institute, Frankfurt, Germany. ^84^African Leadership College (ALC), Pamplemousses, Mauritius. ^85^African Conservation Centre, Nairobi, Kenya. ^86^Department of Economics, Boğaziçi University, Istanbul, Turkey. ^87^University of Grenoble Alpes, IRD, CNRS, INRAE, Grenoble, France. ^88^Centre for Environmental Economics and Policy, School of Agriculture and Environment, University of Western Australia, Perth, Western Australia, Australia. ^89^Global Center for Food, Land and Water Resources, Research Faculty of Agriculture, Hokkaido University, Sapporo, Japan. ^90^Global and International Studies, University of Northern British Columbia, Prince George, British Columbia, Canada. ^91^Nicholaus Copernicus University, Toruń, Poland. ^92^Red de Ecología Funcional, Instituto de Ecología, A.C., Xalapa, México. ^93^German Centre for Integrative Biodiversity Research (iDiv), Leipzig, Germany. ^94^Helmholtz-Centre for Environmental Research (UFZ), Leipzig, Germany. ^95^Cape Horn International Center (CHIC), Universidad de Magallanes, Santiago, Chile. ^96^Department of Biological Sciences and Department of Philosophy and Religion, University of North Texas, Denton, TX, USA. ^97^Northern Research Station, US Forest Service, Evanston, IL, USA. ^98^Aigine Cultural Research Center, Bishkek, Kyrgyz Republic. ^99^School of Geography and Environmental Science, University of Southampton, Southampton, UK. ^100^The Field Museum of Natural History, Chicago, IL, USA. ^101^Ernst & Young ShinNihon LLC, Tokyo, Japan. ^102^National Institute for Environmental Studies, Tsukuba, Japan. ^103^Laboratorio Ecología Humana, Instituto Venezolano de Investigaciones Científicas, Altos de Pipe, Venezuela. ✉**e-mail:** [unai.pascual@bc3research.org](mailto:unai.pascual@bc3research.org)

# **Part A. Introduction to the supplementary information document**

The IPBES *Values Assessment*, a portion of whose data is presented in the main manuscript, was targeted towards a wide range of stakeholders in the public and private sectors and civil society (e.g., governments across administrative scales, multilateral organisations, private enterprise, donors, civil society organisations, Indigenous peoples and local communities, resource managers and users, academia, and media outlets). Data gathering, selection, and analytical approaches were designed to respond to the needs and perspectives of these diverse stakeholders and were iteratively crafted by the team of authors. Some of these stakeholders also participated indirectly in the data gathering and analysis strategies through reviews, dialogues, and workshops undertaken at different stages of the production of the assessment.

Overall, the data sources and assessment strategies differed among the 29 reviews conducted (see details in section B of the Supplementary Information (SI) for each of the review protocols). First, the evidence was **gathered** using combinations of: 1) electronic literature databases (e.g., SCOPUS) and keyword searches, 2) accessible previous reviews (systematic or non-systematic); 3) snow-ball techniques of foundational papers and those identified in (1) and (2); 4) existing theories and typologies; 5) revision of relevant policy repositories (e.g., CBD documents, FAO reports, UNSEEA website, IPBES policy support tools database); 6) calls for contributions from Indigenous and local knowledge experts and knowledge holders; 7) expert knowledge of assessment authors; 8) identification of case studies and associated literature; 9) selection of relevant futures pathways towards sustainability and associated literature; and 10) review of the literature used to support the six chapters of the IPBES *Values Assessment*.

Upon completion of these information-gathering processes, evidence was screened to identify those sources that would be **selected** for in depth analysed, using combinations of: 1) reading title and abstract content; 2) structured sampling of the sources gathered (e.g., by region, random stratified sampling, by research question, per magnitude of environmental impact caused); and 3) reading the full text based on suitability of evidence for the topic analysed within the source.

Evidence determined to be appropriate was subsequently **organised** into databases considering different units of analysis, including 1) each academic publication or document from the grey literature; 2) the study-sites/case studies; 3) the contributions from Indigenous and local knowledge experts and holders; 4) individual futures scenarios; 5) policy instruments; 6) country-specific datasets; and 7) theories.

Finally, evidence was **analysed** in depth, following discipline-specific and type of knowledge-specific standards for each of the 29 review protocols, including: 1) coding of the papers based on readily available typologies, theories, concepts, and research questions; and 2) descriptions of the content of each data source and each category identified. A diversity of quantitative (e.g., frequency analysis, clustering, association coefficients, co-occurrence, parametrization of quantitative indicators or proxies, Bayesian networks, hypothesis testing), qualitative (e.g., content analysis, discourse analysis, selection of excerpts, identification of relationships and similarities, identification of key features, qualitative interpretation), syntheses (e.g., summary tables, figures, narratives, lists), and validation (e.g., quality control revisions) procedures were used, following iterative processes that were sequentially designed and fine-tuned by the team of authors. Input from the three rounds of external reviews and Indigenous and local knowledge and policy dialogues allowed involving a wide range of stakeholders, as well as continued discussions among the author teams, which all enriched these iteration processes.

Diverse approaches to define how evidence is considered and what types of analyses are judged to be robust was explicitly accounted for in designing and undertaking the 29 review protocols. Knowledge and values of stakeholders ranging from those that are place-based and held by Indigenous peoples and local communities (IPLCs) (e.g., farmers, pastoralists, forest managers, and women’s cooperatives) to those derived by regional and global insights by academics from different traditions, the perspectives of policymakers at local to national scales, urban groups and emerging social movements formed around shared values (e.g., neighbourhood associations, youth international movements, landless movements and others) were considered. This diversity is not understood as a dichotomy between IPLCs and Western societies, nor between the Global South and the Global North, nor between Indigenous and local knowledge and academic knowledge, nor even between the social, economic and political sciences and more biophysical sciences, but rather this was undertaken as a multidimensional network of ‘hubs’ or clusters of shared knowledges and values, which may dynamically intertwine and hybridise, like strands in a woven patchwork. Our goal was not to ‘define’ or ‘rank’ these knowledges, but rather seek to bring them together and promote mutual understanding between these ways of understanding and relating to biodiversity and nature.

The assessment approaches used were, nevertheless, limited by the lack of accessibility to some academic and non-academic literature in languages other than English, the lack of capacity of experts to equally review literature in diverse languages, the differing perspectives on what is considered valid evidence, and the diverse reporting formats across disciplines. The capacity of this co-author team to perceive, represent, and assess the diversity of values that have guided and informed the decisions and actions of individuals, families, communities, and others since time immemorial is bounded by the conditions that underpin the production of the assessment. The approaches used and insights gained have been influenced by the broader framing of the IPBES conceptual framework, IPBES rules of procedure (e.g., the use of literature reviews) and structures (e.g., the disciplinary or regional representation and organisation of experts) that have guided the *Values Assessment* (see: IPBES *Guide on the Production of Assessments* (<https://doi.org/10.5281/zenodo.7568075>). Consequently, only a portion of the vast spectrum of humanity’s diverse perspectives can be reflected here. For example, grey literature can be difficult to identify using search engines. In particular, some governmental documents (e.g., policies, laws) are not easily accessible, which explains why they only represented a small fraction of the sources in this assessment (11%), despite devoting significant effort to avoid this bias.

# **Part B. Overview of reviews and review protocols**

**Table SI.B0:** Overview of reviews and review protocols

| **Feature** | **Description** | **Total number of reviews** | **Information Document (#)** |
| --- | --- | --- | --- |
| **Types of reviews (some reviews used more than one type of review)** | 1. Comprehensive structured review[1] | 5 | 4, 9, 10, 19, 29 |
|  | 2. Semi-structured review partially based on expert criteria[2] | 10 | 3, 5, 6, 7, 11, 14, 15, 22, 23, 24 |
|  | 3. Non-structured review fully based on expert criteria[3] | 2 | 25, 27 |
|  | 4. Invited contributions from external experts and stakeholders[4] | 2 | 8, 18 |
|  | 5. Combinations of the above | 10 | 1, 2, 12, 13, 16, 17, 20, 21, 26, 28 |
| **Type of source (some reviews used more than one type of source)** | 1. Peer-reviewed literature (including scientific papers and books) | 26 | 1, 2, 3, 4, 5, 6, 9, 10, 11, 12, 13, 14, 15, 16, 17, 18, 19, 20, 21, 22, 23, 24, 25, 26, 28, 29 |
|  | 2. Grey literature | 15 | 2, 5, 7, 12, 13, 15, 16, 17, 18, 22, 23, 25, 26, 28, 29 |
|  | 3. Contributions from invited experts | 6 | 8, 13, 16, 17, 18, 26 |
|  | 4. Other (chapters of the IPBES *Values Assessment*; pieces of art; conferences & webinars; journalistic pieces) | 4 | 13, 26, 27, 28 |
| **Process to gather evidence (some reviews used more than one type of process)** | Keyword search in academic datasets | 24 | 1, 2, 4, 5, 6, 9, 10, 11, 12, 13, 14, 15, 16, 17, 18, 19, 20, 21, 22, 23, 24, 26, 28, 29 |
|  | Literature selected through expert knowledge | 11 | 2, 3, 7, 12, 15, 22, 23, 24, 25, 26, 28 |
|  | Information gathered from other experts | 9 | 8, 13, 16, 17, 18, 21, 26, 27, 28 |
|  | Snow-ball technique | 2 | 2, 23 |
|  | Search in the web | 3 | 13, 23, 29 |
| **Type of analysis** | Qualitative | 16 | 2, 5, 6, 7, 11, 13, 16, 17, 18, 19, 20, 21, 25, 26, 27, 29 |
|  | Quantitative | 3 | 4, 9, 10 |
|  | Qualitative & quantitative | 10 | 1, 3, 8, 12, 14, 15, 22, 23, 24, 28 |
| **Section of the paper the review was undertaken (one review could have informed several sections)** | 2. The values of nature are diverse | 11 | 1, 2, 3, 4, 5, 6, 13, 15, 19, 21, 24 |
|  | 3. Valuation methods are diverse and spreading but uptake remains limited | 9 | 8, 9, 10, 11, 13, 14, 19, 21, 22 |
|  | 4. Considering diverse values improves decision outcomes | 9 | 1, 12, 13, 16, 17, 18, 19, 20, 21 |
|  | 5. Leveraging nature's values for transformative change | 6 | 19, 21, 24, 26, 27, 28 |
|  | 6. The diversity of values of nature underpin multiple pathways towards sustainability | 3 | 19, 23, 25 |
|  | 7. Shifts are needed in values and valuation to foster transformative changes | 7 | 1, 3, 7, 19, 24, 26, 29 |
|  | 8. Conclusions | 2 | 19, 24 |

## **B1. Review to develop the typology of “specific” values of nature**

**Table SI.B1:** Summary of review to develop the typology of “specific” values of nature

| **Specific topics supported:**  Typology of “specific” values of nature | **Type of review:**  Combination of reviews:  ● Comprehensive structured review  ● Semi-structured review partially based on expert criteria  ● Non-structured review fully based on expert criteria |
| --- | --- |
| **DOI of associated data management report:**  10.5281/zenodo.4396289 | **Type of sources:**  Peer reviewed literature |
| **Section of the paper where this is covered:**  Sections 1, 3, and 6 | **Language(s) in which the review was carried:**  Search string was in English. Other papers retrieved were in English (the majority), Spanish, Portuguese, Polish, Russian and German. |

**Purpose of gathering the evidence:**

There were three main purposes of the review: i) to identify how the specific value types adopted in the IPBES Conceptual Framework (intrinsic, instrumental, relational values) are directly defined or indirectly described/ understood in the literature across disciplines; ii) to identify reference to, definition of, and salient associations with other relevant concepts, such as worldviews and knowledge systems, understanding of human-nature relationships, and the role of institutions in value formation and change; and iii) to extract information about framing (prioritisation or obscuration of particular values or knowledge systems) and about relevance for policy

**Process followed to gather the evidence:**

The evidence was gathered via a literature search in different databases (to cover different disciplines), using Boolean operators[5] combining each of the value types indicated in former IPBES assessments (intrinsic, instrumental, relational) with other key terms, such as "ecosystem services", "Nature Contribution to People", or more general terms such as "natur*" or "environment*". The databases searched were Web of Science; Scopus; Google Scholar; EBSCOhost (Academic Search Premier)

The information of the papers was collected in .csv files and numbered. After a first analysis of results, 32 additional papers were added based on expert knowledge (core publications in the field, sometimes in book collections and therefore not appearing in databases).

**Process to screen the information gathered:**

The references found were screened based on title and abstract in order to eliminate duplicates and non-relevant documents (for example, documents not referring to environmental issues or where environmental issues were not central, but only mentioned as examples to illustrate a concept).

**Unit of the analysis:**

Academic papers or chapters

**N(initial - gathered):**

683 academic papers

**N(final - analysed):**

239 academic papers

**How was the evidence organised:**

The retrieved papers were organised in a spreadsheet table that included bibliographic information of each paper, the names of the reviewers that were to code each paper and the 14 codes[6] used to analyse them, which were about specific values (intrinsic, instrumental and relational), prominent ecosystem valuation concepts (ecosystem services, natural capital, nature’s contributions to people, among others), knowledge systems, policy applications of these concepts, human-nature relationships, types of worldviews, type of study, institutions and values they prioritise, and types/ways of values being obscured or neglected.

**Description of the analysis of the evidence:**

Evidence was analysed on the ground of the 14 codes, both quantitatively (code present/absent) and qualitatively (how addressed in the paper). Each code was described in detail to secure common interpretation across different analysts. In the spreadsheet each code occupied one or more (for sub-categories) columns.

A sub-review of one of the codes (number 13 of the list above) allowed reviewers to identify papers addressing the issue of framing in environmental values and valuation. When coders determined that a manuscript addressed the issue of framing, they then summarised (in 1-5 sentences) what the article said about framing. These summaries were then analysed using thematic coding techniques. NVivo 12 software was employed.

## **B2. Review on value formation and change**

**Table SI.B2:** Summary of review on value formation and change

| **Specific topics supported:**  Value formation and change (including in the face of natural disasters and extreme events) | **Type of review:**  Combination of reviews:  ● Comprehensive structured review  ● Semi-structured review partially based on expert criteria  ● Non-structured review fully based on expert criteria |
| --- | --- |
| **DOI of associated data management report:**  Partially based on 10.5281/zenodo.4071755 | **Type of sources:**  Peer reviewed literature and grey literature |
| **Section of the paper where this is covered:**  Section 1 | **Language(s) in which the review was carried:**  English, Spanish |

**Purpose of gathering the evidence:**

To describe the ways (or ‘how’) values form and change and gain perspective on the understanding of these processes across disciplines and knowledge traditions; it did not aim to assess quantitative trends in publication patterns. These changes also were explored explicitly for the case of natural disasters and extreme events.

**Process followed to gather the evidence:**

This assessment used a multi-staged scoping review to identify and describe a range of disciplines and associated concepts involved in explaining how values form and change, including in the face of natural disasters and extreme events. The review was structured based on the typology of Kendall and Raymond (2019)[7] that delimits individual, social and social-ecological mechanisms of values formation and change. Evidence was gathered from a systematic review[8], snow-ball techniques, and finally to complement the previous sources, five authors conducted individual expert-driven searches in academic databases (e.g., Web of Science, Scopus, Google Scholar) to ensure disciplinary representation. Explicit searches were made within the areas of anthropology, economics, education, environmental education, natural disasters and extreme events, philosophy, psychology, religion and sociology.

**Process to screen the information gathered:**

The first screening entailed a comprehensive structured review[9] carried out more broadly for chapter 2 of the IPBES *Values Assessment*[10], which included a code for 'values formation/change' (n=94, which was 20% of the total articles in the review). These publications (all of which were academic review articles) were then read by two authors to determine their relevance and whether they cited other references (i.e. a 'snow-ball technique') that would fit within the review's objectives and to determine seminal sources or relevant publications before the systematic review's 2005 begin date (n=22).

**Unit of the analysis:**

Documents (considering peer-reviewed and grey literature)

**N(initial - gathered):**

232 documents

**N (final - analysed):**

133 documents

**How was the evidence organised:**

Information was organised into the structured rubric provided by Kendal and Raymond (2019) and presented as individual, social and social-ecological mechanisms of values formation/change. Additionally, information on natural disasters and extreme events was synthesised to highlight this topic. While there was abundant information on how these events affect values (e.g., hectares of mangrove or dollars to reconstruct), there was a relative dearth on how they affect values formation/change.

**Description of the analysis of the evidence:**

Evidence was analysed to describe the mechanisms that underlie the different concepts of values formation/change. It was also used to highlight similarities and differences between different disciplines or perspectives. Finally, information was synthesised in a table to present a summary of the concepts and their perspective on this topic. It was also depicted graphically in a figure that related these concepts (form/change) with values expression (the previous section of the chapter).

## **B3. Review on behavioural theories and their links to values**

**Table SI.B3:** Summary review on behavioural theories and their links to values

| **Specific topics supported:**  Link between values and behaviour | **Type of review:**  Semi-structured review partially based on expert criteria |
| --- | --- |
| **DOI of associated data management report:**  10.5281/zenodo.4399396 | **Type of sources:**  Peer-reviewed literature |
| **Section of the paper where this is covered:**  Sections 1, 6 | **Language(s) in which the review was carried:**  English |

**Purpose of gathering the evidence:**

To understand the role that values play in prominent theories of human behaviour and categorising the roles that values and value-related constructs play in each theory.

**Process followed to gather the evidence:**

Two existing and widely-cited reviews of behaviour theories were used to generate a list of 144 theories in total. The two reviews emerged from different disciplinary contexts; each refined the lists of theories they address in different ways. One assembled theories based on expert knowledge (Michie et al., 2014)[11]. The other (Kwon and Silva, 2020)[12] was based on a systematic search (all databases on Web of Science) of the term “behaviour,” followed by selection of theories for analysis based on citation counts (those with the most citations, normalised for publication date, were included) .

**Process to screen the information gathered:**

All behavioural theories included in either of the two reviews were analysed (duplicates were eliminated, but no theory was excluded).

**Unit of the analysis:**

Theory

**N(initial - gathered):**

144 theories (the sum of all the theories covered in both reviews). Ten theories appeared in both reviews, but duplicates were removed, leaving 134 theories

**N(final - analysed):**

134 theories

**How was the evidence organised:**

For each theory, the following data were recorded: theory name, author, year of publication, every construct included in the theory (i.e., all factors the theory identified as associated with behaviour), a description of each construct, whether the construct was value-related or not, the categorization of all value-related constructs (e.g., broad value, specific value, value-adjacent). Theories listed in Michie et al. 2014 were already associated with lists of constructs that wereused for this analysis. Theories listed only in Kwon and Silva had no lists of constructs; reviewers engaged in a detailed, multi-step coding process to identify constructs that were consistent with the Michie et al approach.

**Description of the analysis of the evidence:**

A total of 2,232 constructs were analysed to determine whether they were value-related -- i.e., whether they constituted a broad or specific value. Those that constituted a broad or specific value were coded into categories of broad and specific values. Then, a quantitative analysis was conducted to assess: i) the number and types of value-related constructs; ii) how value-related constructs co-occurred; iii) which disciplines were most represented in the review; and iv) whether there were patterns in the use of value-related constructs over time.

## **B.4. Review on the conceptualizations of nature’s values**

**Table SI.B4:** Summary of review on the conceptualizations of nature’s values

| **Specific topics supported:**  Conceptualizations of nature’s values | **Type of review:**  Comprehensive structured review |
| --- | --- |
| **DOI of associated data management report:**  10.5281/zenodo.4071755 | **Type of sources:**  Peer-reviewed literature |
| **Section of the paper where this is covered:**  Section 1 | **Language(s) in which the review was carried:**  English, German, French, Italian, Dutch and Spanish |

**Purpose of gathering the evidence:**

To examine the associations between nature's diverse values and the four life frames of nature’s values.

**Process followed to gather the evidence:**

A search was conducted in Scopus database for articles between January 1, 2005 and May 16, 2019 using the search strings “valu*” (i.e., value, values, valuation etc.), “wellbeing”, or “quality of life”, combined with “nature”, “ecosystem*”, “biodiversity” or “landscape”), and additionally for environmental “valu*”, “human-nature relat*” or society-nature relat*”. The time period was chosen to coincide with the year of publication of the Millennium Ecosystem Assessment. Because these searches retrieved over 100,000 publications, the search was restricted to review papers and editorials, which yielded 7,204 results.

**Process to screen the information gathered:**

All results were screened by two researchers on the basis of title and source; if one of the researchers included an article, this was included in the sample for full text screening; this provided 681 English language results and 16 results in other languages. A total of 697 sources were considered for full text review. From these, 102 full texts could not be acquired, one Hungarian article was excluded due to a lack of language capacity within the assessment team, and 122 sources were excluded from quantitative analysis following full-text review because they fell outside of the scope or because they were not review articles or editorials. In the latter case, they could still be drawn on qualitatively. This left a final sample of 496 articles that were coded using 55 different codes, including codes for the four life frames (i.e. living from, with, in and, as nature). The first 50 articles were coded and assessed for intercoder reliability using the Kappa coefficient, returning 0.61, indicating substantial agreement. The pilot sample was re-coded and again analysed for reliability, returning a Kappa of 0.94, which suggests near complete agreement.

**Unit of the analysis:**

Papers

**N(initial - gathered):**

7,204 papers

**N(final - analysed):**

496 papers

**How was the evidence organised:**

Data were organised in MS Excel Sheet, which was then inputted into SPSS. The spreadsheet contained basic bibliographic information for each of the papers, as well as one column per each of the 55 codes[13] used to review the information, which related to general topics such as: indigenous and local knowledge, value types, values and institutions, values and decision-making, and value formation and change.

**Description of the analysis of the evidence:**

To shed light on the association between certain codes identified in the literature, two approaches were used. The first was hierarchical cluster analysis. SPSS 27 was used to assess clusters between variables, using Ward’s linkage method based on Euclidean distances. A second approach involved estimating coefficients of association: Phi and Yule’s Q coefficients were generated using SPSS 27. The Yule’s Q coefficient is a derivation of Kruskal’s Gamma, indicating the strength of relationship between dichotomous variables[14].

## **B5. Review on value articulating institutions**

**Table SI.B5:** Summary of review on value articulating institutions

| **Specific topics supported:**  Value articulating institutions | **Type of review:**  Semi-structured review partially based on expert criteria |
| --- | --- |
| **DOI of associated data management report:**  10.5281/zenodo.4399373 | **Type of sources:**  Peer-reviewed literature and grey literature |
| **Section of the paper where this is covered:**  Section 1 | **Language(s) in which the review was carried:**  English |

**Purpose of gathering the evidence:**

To explore i) conceptual approaches on how institutions (rules, norms, rights) influence the expression of values and ii) empirical assessments on how the inherent rules and procedures of environmental valuation methods and policies influence the expression of values.

**Process followed to gather the evidence:**

Search in Google Scholar. The terms used were: TITLE-ABS-KEY (Value Articulating Institutions); TITLE-ABS-KEY (cost-benefit analysis AND distributional weights) OR (cost-benefit analysis AND multicriteria analysis); TITLE-ABS-KEY (integrated OR plural) AND (valuation); TITLE-ABS-KEY (reflexivity AND valuation)

**Process to screen the information gathered:**

The retrieved references were reviewed by the project leader. Based on expert knowledge, relevant information was selected using titles and abstract.

**Unit of the analysis:**

Academic papers

**N(initial - gathered):**

86 papers

**N(final - analysed):**

16 papers

**How was the evidence organised:**

A database was created to code the relevant papers according to the following general topics: methods applied; value conflicts; participation forms; participants; value expression forms; communications among participants; assumption: values as given; incommensurability; knowledge systems; value types; reflexivity about the appropriateness of the method, influence of measurement systems and valuation processes on conclusions and recommendations; power relations.

**Description of the analysis of the evidence:**

Specific topics to analyse the information were: i) what is the method addressed by the paper and what sort of values and classifications analyses (monetary values , biophysical values, sociocultural values, health related values, indigenous values, traditional and local knowledge, others); ii) methods used in the assessment (unique method or integrative); iii) how integration was produced; iv) the paper considers how the valuation approach and method faces and manage value conflict; v) the paper considers who should participate, and what role and competence the participants shall play (as consumers, citizens, stakeholders, experts); vi) the paper considers the implications of power relation among participant; vii) the paper considers the implications of how the participants are selected or invited to the valuation; viii) the paper consider how are participants supposed to participate – individually and/or as part of a group; ix) the paper consider how the value expression forms (in writing or orally, virtual others), influence the results; x) the paper considers the implications of communication between participants on the value outcomes; xi) the paper consider the role of values as given, or as a result from the valuation process (value change); xii) the paper considers the power relations associated to valuation processes; xiii) the paper considers which valuation method and approach (institutional structure) is best suited for treating the valuation aim (issue at hand); xiv) the paper discusses how valuation methods face complexity and uncertainty; xv) the paper considers what knowledge systems are relevant for the assessment; xvi) what is said about the typologies of value that are excluded or included in the valuation, eg biophysical, sociocultural, economic, local, indigenous, other, intrinsic, relational or instrumental values; xvii) the paper considers the incommensurability of values; xviii) the paper considers how measurement systems affect the valuation outcomes (prices, weights, arguments, statements); xix) the paper considers how data shall be produced (norms or principles required to produce these data) according of the unit of analysis; xx) the paper considers how the conclusions shall be produced and how methods influence the conclusions; xxi) the paper considers how the power relations may affect the production of conclusions and recommendations; xxii) the paper considers if elements external to the technical and academic processes of valuation (corruption, political pressure), may influence the results; xxiii) the paper produces recommendations on how to develop and improve the valuation.

## **B.6. Review of norms, rules and rights relative to the valuation context**

**Table SI.B6:** Summary of review of norms, rules and rights relative to the valuation context

| **Specific topics supported**  Norms, rules and rights relative to the valuation context | **Type of review:**  Semi-structured review partially based on expert criteria. |
| --- | --- |
| **DOI of associated data management report:**  10.5281/zenodo.4071755 | **Type of sources:**  Peer-reviewed literature |
| **Section of the paper where this is covered:**  Section 1 | **Language(s) in which the review was carried:**  English |

**Purpose of gathering the evidence:**

To explore how the relation between institutions and values is addressed in different academic fields and management contexts, to identify, characterise and assess, the different conceptualizations of nature and its multiple values, including human-nature relationships, from different academic and socio-cultural traditions and perspectives across review papers.

**Process followed to gather the evidence:**

The initial sample was obtained from applying the following search string in Scopus: TS= (TITLE-ABS-KEY( ((valu* OR wellbeing OR well-being OR "quality of life") AND (nature OR ecosystem OR biodiversity OR landscape) OR "environmental valu*" OR "human-nature relat*" OR "society-nature relat*") AND (DOCTYPE(ed) OR DOCTYPE(re)) AND PUBYEAR > 2004 AND SUBJAREA (ARTS OR BUSI OR DECI OR ECON OR PSYC OR SOCI OR ENVI OR EARTH OR MULT OR Undefined)

**Process to screen the information gathered:**

The structured review on conceptualizations of values had a code for “norms, rules, rights, relative to the institutional context of valuation”. Those records that presented evidence for this code were revisited for the purpose of this review based on expert criteria.

**Unit of the analysis:**

Academic papers

**N(initial - gathered):**

268 papers

**N(final - analysed):**

228 papers

**How was the evidence organised:**

A database was created to add the information obtained from revisiting and recoding the papers.

**Description of the analysis of the evidence:**

The papers were re-coded and analysed along the following general topics: i) description of the institution at stake and the values and life frames articulated; ii) value plurality; iii) rationalities emphasized by the institution; iv) conflicts and power relations related to the institution; v) valuation methods and reflexivity; vi) procedures about how decisions and consultation should be made; and vii) assumptions on behavioural change and policy support.

## **B.7. Review of values embedded into biodiversity and sustainability policy documents**

**Table SI.B7:** Summary of review of values embedded into biodiversity and sustainability policy documents

| **Specific topics supported:**  Types of values embedded in policy documents | **Type of review:**  Semi-structured review partially based on expert criteria |
| --- | --- |
| **DOI of associated data management report:**  10.5281/zenodo.4399907 | **Type of sources:**  Grey literature |
| **Section of the paper where this is covered:**  Section 6 | **Language(s) in which the review was carried:**  English, Spanish |

**Purpose of gathering the evidence:**

To assess how values are represented in policy documents from the Global North and Global South, including National Biodiversity Strategies and Action Plans, the Millennium Ecosystem Assessment, reports from The Food and Agriculture Organization (FAO), the Intergovernmental Science-Policy Platform on Biodiversity and Ecosystem Services (IPBES) and other relevant texts from international organisations.

**Process followed to gather the evidence:**

Different policy-relevant ‘domains’ were considered based on expert knowledge (e.g., CBD, IPBES, FAO, SDGs and other major global and regional reports). For CBD documents, those related to the Aichi and post2020 targets (n=2), and 16 randomly selected National Biodiversity Strategies and Action Plans, NBSAPs (8 from Global South and 8 from Global North), were used. This was complemented with 2 reports related to the 2030 Agenda for Sustainable Development and all previously approved IPBES summaries for policymakers (n=8). Two regional agreements (the Biodiversity Strategy for the European Union and the Latin American Escazú Agreement) were incorporated, as well. Furthermore, 5 FAO reports provided a perspective from forestry, fisheries and agriculture. Finally, the WWF *Living Planet Report*, the *Dasgupta Review*, The Economics of the Environment and Biodiversity, and the *Millennium Ecosystem Assessment*, were included for review.

**Process to screen the information gathered:**

Documents were selected based on criteria defined by the experts regarding their relevance for the review. All selected documents were reviewed.

**Unit of the analysis:**

Policy document

**N(initial - gathered):**

37 documents

**N(final - analysed):**

37 documents

**How was the evidence organised:**

A spreadsheet was created to register coded information for each policy document. The documents were coded following three general topics (Policy document type/context; Knowledge system/worldviews; General themes), for a total of 22 codes[15].

**Description of the analysis of the evidence:**

Documents were read by three coders. Coding was carried out as a form of qualitative content analysis. This began with the formation of the codebook that was used to guide the coding process, which was based on a combination of responding to key messages and themes (e.g., approach to justice) from chapter 2 of the IPBES *Values Assessment*[16], as well as drawing upon existing conceptual frameworks (e.g., life frames) to guide the analysis. In terms of the coding itself, the majority of the codes entailed descriptive content as opposed to a pre-defined typology or data range. This descriptive content was either direct references from the data itself, or were summarised statements from the coders’ own understandings. To ensure quality control during the coding process, the three coders regularly discussed and communicated individual processes and methods for coding the documents. As a result, the data was constructed through an iterative process, whereby changes were made to the codebook as emerging themes developed. Retrieved data was synthesised by describing the findings for each of the codes by policy ‘domain’.

## **B.8. Contributions on values and valuation methods by Indigenous Peoples and local communities (IPLCs)**

**Table SI.B8:** Summary of contributions on values and valuation methods by Indigenous Peoples and local communities (IPLCs)

| **Specific topics supported:**  Nature valuation by Indigenous peoples and local communities (IPLCs) | **Type of review:**  Invited contributions from external experts and stakeholders |
| --- | --- |
| **DOI of associated data management report:**  10.5281/zenodo.4404612 | **Type of sources:**  Contributions written by summoned experts |
| **Section of the paper where this is covered:**  Section 2 | **Language(s) in which the review was carried:**  English, Spanish |

**Purpose of gathering the evidence:**

To gather information about the use of nature valuation in Indigenous peoples and local communities (IPLCs) contexts and by IPLC.

**Process followed to gather the evidence:**

Contributions were sought from experts on Indigenous and local knowledge (ILK) and Indigenous and local knowledge holders. Invitations were sent to a total of 76 experts spanning different regions of the globe and livelihood types. Experts were identified from their publication records and by asking for recommendations from authors of the IPBES values assessment and the IPBES taskforce on ILK. Potentially interested contributors were sent a set of questions regarding i) background information about the indigenous people or local community (location, sources of livelihood); ii) description of the socio-political context; iii) description of the way valuation is conducted within the community or by the group; and iv) how the results of the valuation are shared or made public. Interested contributors could respond to these questions directly or in the form of an essay, providing references to supporting evidence when possible.

**Process to screen the information gathered:**

Only excerpts responding directly and indirectly to the questions explicitly related to nature valuation were included in the analysis.

**Unit of the analysis:**

Essays

**N(initial - gathered):**

26 essays

**N(final - analysed):**

26 essays

**How was the evidence organised:**

Each essay was saved in an individual text file and uploaded to the web-based app Dedoose. A tree of codes was generated and applied to the relevant text.

**Description of the analysis of the evidence:**

For each valuation-related question, relevant excerpts were consolidated and analysed (within Dedoose) using descriptive statistics to identify patterns and trends across the sample size. Frequencies of code application across a range of IPLC attributes (such as livelihood type, region, worldview) were computed to generate lists of valuation approaches identified in the texts and to detect conditions for valuation in IPLC contexts including types of values elicited and purposes of valuation. A complementary approach based on qualitative interpretation of the data by ILK holders (authors of the assessment) was applied to the same data to demonstrate how a different analytic approach might better reflect IPLC worldviews. Approaches were compared to identify the values and limitations of integrating western and IPLC knowledge systems to understand IPLC valuation.

## **B.9. Review of valuation methods and approaches**

**Table SI.B9:** Summary of review of valuation methods and approaches

| **Specific topics supported:**  Valuation methods and approaches | **Type of review:**  Comprehensive structured review |
| --- | --- |
| **DOI of associated data management report:**  10.5281/zenodo.6468906 | **Type of sources:**  Peer-reviewed literature |
| **Section of the paper where this is covered:**  Section 2 | **Language(s) in which the review was carried:**  English |

**Purpose of gathering the evidence:**

Firstly, to establish how widespread nature valuation is globally and how the research field has developed over time. Secondly, to identify the abundance of different types of valuation methods and analyse whether abundance of valuation at the country scale correlates with economic, social and environmental variables.

**Process followed to gather the evidence:**

The search is based on overlapping three sets of papers registered in Web of Science. The three sets were identified using search terms to select i) papers on nature and biodiversity; ii) methods (divided in search terms for the four methods families); iii) value dimensions. The overlap of the sets identifies the corpus for the review.[17]

**Process to screen the information gathered:**

The papers in the corpus were georeferenced from country names in titles, abstracts, and key words. ISO 3 CODES were added to the database using ISO 3166 country codes. Papers with no information on where the study was conducted were excluded from the analysis.

**Unit of the analysis:**

Papers

**N(initial - gathered):**

79,040 papers

**N(final - analysed):**

48,781 papers

**How was the evidence organised:**

A database with information on the country the valuation case study was conducted, year of publication, valuation method family applied was built. This was linked to country level data on indicators of economic development, demographic and environmental pressure indicators.

**Description of the analysis of the evidence:**

To identify applications of valuation methods and relate them to their social, environmental and political contexts, we selected a subset of papers (48,781) that could be georeferenced based on country names in titles, abstracts and keywords. The density of valuation studies per country was correlated with social and biological indicators including IPBES core indicators such as the human development index (HDI), average harmonised learning outcomes score, gross domestic product (GDP), corruption perception index (CPI), and population size. Correlations between valuation densities and the indicators were analysed using Pearson correlation statistics and testing for robustness of findings replicating the analysis using log transformations.

## **B.10. Review of valuation methods based on principles, criteria, indicators and verifiers**

**Table SI.B10:** Summary of review of valuation methods based on principles, criteria, indicators and verifiers

| **Specific topics supported:**  Valuation methods: principles, criteria, indicators and verifiers | **Type of review:**  Comprehensive structured review |
| --- | --- |
| **DOI of associated data management report:**  10.5281/zenodo.4404678 | **Type of sources:**  Peer-reviewed literature |
| **Section of the paper where this is covered:**  Section 2 | **Language(s) in which the review was carried:**  English |

**Purpose of gathering the evidence:**

To assess which societal goals valuation seek to achieve, in which environmental and societal contexts are they used; which valuation methods and approaches are used to assess different types of values; how values are expressed using methods; which stakeholder's values are assessed; and how diverse values are combined to inform decision-making. An additional analysis looked specifically at documented uptake of economic valuation methods compared to other methods, to assess the hypothesis that the uptake of results using economic methods have been documented to a larger extent than uptake of other methods.

**Process followed to gather the evidence:**

A Web of Science search[18] was the basis for this review. From this corpus papers focused on supporting decision-making were identified using search terms[19]. This retained 33,248 papers for further screening. The papers selected for full paper review were sampled from this reduced corpus using random stratified sampling of papers published in the period 2010-2020, stratified based on the four global regions of IPBES[20] and to cover the methods families (see also SI #9).

**Process to screen the information gathered:**

The random stratified sample of papers were screened based on experts reading of abstracts. Papers reporting on results from applications of valuation methods to make values of nature visible in decision making were retained for full paper review.

**Unit of the analysis:**

Applications of valuation methods and approaches within papers. One same paper could report on the application of a method in more than one study site counting as more than one application.

**N(initial - gathered):**

79,040 papers

**N(final - analysed):**

1,163 valuation applications

**How was the evidence organised:**

The final set of papers were divided between coders and information coded for each of the applications was recorded in an online form, which allowed generating a spreadsheet with information on how the valuation methods were applied (see further detail below).

**Description of the analysis of the evidence:**

Using test rounds, a coding manual was developed to code the full content of the papers according to 474 questions organized around 8 topics: 1) methods and their use, 2) context of application, 3) application descriptors, 4) reliability and validity, 5) Indigenous peoples and local communities, 6) human well-being, 7) ecological sustainability and 8) justice. The coding was undertaken following standardised procedures which included confidence statements. A central database with answers to the 474 questions, mainly closed (yes/no) questions, was the basis for quantitative analyses. Analyses focused on characterising differences between methods and method families. Findings based on analysis of the sample were corrected for the abundance of published studies across IPBES regions and method families in the initial sample. The reported statistics therefore represent 'global valuation practice'. To assess the uptake of economic methods, all economic valuation methods, except decision-support tools, were grouped and compared to other valuation methods in terms of level of documented uptake.

## **B.11. Review of valuation method families**

**Table SI.B11**: Summary of valuation method families

| **Specific topics supported:**  Valuation method families | **Type of review:**  Semi-structured review partially based on expert criteria |
| --- | --- |
| **DOI of associated data management report:**  10.5281/zenodo.4404436 | **Type of sources:**  Peer reviewed literature |
| **Section of the paper where this is covered:**  Section 2 | **Language(s) in which the review was carried:**  English, Spanish |

**Purpose of gathering the evidence:**

To group and characterise valuation methods and approaches into method families.

**Process followed to gather the evidence:**

Four individual searches were conducted, using Web of Science, Scopus and ScienceDirect, which were later complemented with textbooks and technical valuation manuals.

Each of the searches corresponded to each of the main valuation method family and search terms used are as follows: i) The search for papers on nature based valuation methods used combinations of search terms like “biophysical”, “valuation”, ”mapping”. Ii) The search for papers on statement-based valuation methods used search terms like “Deliberative valuation”, “stated preference*”, “Q-methodology”. iii) The search for papers on behaviour-based valuation methods used search terms like "Revealed preference*", “damage cost”, “Livelihood assessment”. iv) The search for papers on integrated valuation methods used search terms like “multi criteria” AND “decision making”, “integrated modelling”, “Participatory mapping”, “group deliberation”. The full list of search terms can be found in 10.5281/zenodo.4404436.

**Process to screen the information gathered:**

Duplicates were eliminated. Titles were screened for relevance; non-relevant documents were omitted. Then, abstracts of remaining documents were screened for relevance (describing a method or sets of methods, reviews of methods, reviews of strengths and limitations of methods), again, non-relevant documents were deleted. Finally, full texts were screened for relevance; relevant text was highlighted and texts without relevant information were omitted.

**Unit of the analysis:**

Papers (on valuation methods)

**N(initial - gathered):**

3,557 papers

**N(final - analysed):**

420 papers

**How was the evidence organised:**

Basic bibliographic information of the selected samples were kept in spreadsheets. Full text of the most relevant sources was retrieved to be fully read.

**Description of the analysis of the evidence:**

The literature and web resources were screened for lists of individual methods and approaches and their classification. The list was complemented with additional methods based on expert input. Methods were divided into four broad methods families based on their source of value information. Each method was characterised, and their scope, strengths and limitations were identified.

## **B.12. Review of values and motivational crowding by economic incentives in conservation policy**

**Table SI.B12:** Summary of review of values and motivational crowding by economic incentives in conservation policy

| **Specific topics supported:**  Links between values and motivations for conservation under economic incentives | **Type of review:**  Combination of reviews:  ● Comprehensive structured review  ● Semi-structured review partially based on expert criteria |
| --- | --- |
| **DOI of associated data management report:**  10.5281/zenodo.4390995 | **Type of sources:**  Peer reviewed literature and grey literature |
| **Section of the paper where this is covered:**  Section 3 | **Language(s) in which the review was carried:**  English |

**Purpose of gathering the evidence:**

To examine whether the introduction of economic incentive policies affects intrinsic values and motivations for nature conservation, and which policy design features matter for the answer. This review uses the terms of crowding in (when a situation or intervention increases a given behaviour) and crowding out (when a situation or intervention drives down a given behaviour).

**Process followed to gather the evidence:**

Three sources of information were used:

i) Inclusion of the papers reviewed by Rode et al. (2015)[21]; ii) search in Web of Science (search terms: (motivation*) AND (crowd*) AND (environment* OR biodiversity OR ecolog* OR ecosystem OR forest OR “land use” OR agricultur* OR conservation*) AND (incentive* OR payment* OR polic* OR economic* OR compensate* OR mone* OR reward*) AND (experiment* OR survey* OR interview* “field stud*” OR “case stud*” OR workshop* or questionnaire*))) AND LANGUAGE: (English) AND DOCUMENT TYPES: (Article); iii) incorporation of additional literature through expert knowledge and cross-check with studies listed in other major review articles.

**Process to screen the information gathered:**

Duplicates from different stages of the review were screened out. Then, abstracts of all articles were screened to identify if they satisfied the pre-defined criteria: i) presents an empirical study; ii) has a clear link to economic/incentive-based conservation policies; iii) explicitly discusses motivation crowding (this can be expressed under a different name).

**Unit of the analysis:**

Academic articles

**N(initial - gathered):**

116 papers (including 8 duplicates)

**N(final - analysed):**

69 papers

**How was the evidence organised:**

A spreadsheet was created, which included, among others, the following fields: type of study (e.g. experiment, case study), sample characteristics and location, outcome measure (behaviour or motivations), whether studies measured motivation crowding during and/or post policy, the finding regarding motivation crowding, various study design features. A text document was used to add studies analysing different policy designs, containing policy design features and variants analysed, and results on motivation crowding by variant.

**Description of the analysis of the evidence:**

First, the general evidence for crowding out vs. crowding in vs. no effect was analysed, but it was concluded that it was not meaningful to count observations due to publication bias. Insights were drawn on methodological differences and weaknesses of studies. Then, for the 32 studies analysing different policy design variants, it was analysed whether the study's evidence was methodologically sound (studies suffering from self-selection bias were considered not to be). Twelve policy features were identified, for which studies had analysed different variants and how these affect motivation crowding. For policy features where at least two sound studies analysed relative motivation crowding for similar variants, findings were reviewed on how the variants compared in terms of motivation crowding, and - if results differed -, possible influencing factors.

## **B.13. Review of values considered in local decision-making contexts about nature**

**Table SI.B13**: Summary of review of values considered in local decision-making contexts about nature

| **Specific topics supported:**  Values of nature considered in decision-making at the local level under asymmetric power relations | **Type of review:**  Combination of reviews:  ● Comprehensive structured review  ● Semi-structured review partially based on expert criteria  ● Non-structured review fully based on expert criteria  ● Invited contributions from external experts and stakeholders |
| --- | --- |
| **DOI of associated data management report:**  10.5281/zenodo.4396271 | **Type of sources:**  Peer reviewed literature, grey literature, and other (pieces of art; conferences & webinars; contributions of relevant stakeholders) |
| **Section of the paper where this is covered:**  Sections 1, 2, 3 | **Language(s) in which the review was carried:**  English, Spanish |

**Purpose of gathering the evidence:**

To provide an example (in this case, of the Amazon) to assess how economic and political processes shape power relations among value types that constrain access to nature.

**Process followed to gather the evidence:**

Evidence was gathered in multiple steps:

i) search conducted in Google search engine and bibliographical global databases [CBD, FAO, UNEP, UN Sustainable Development, UNESCO] to identify and select policy instruments (legislations; judicial decisions; chapters, articles and laws of two national Constitutions; court rulings; declarations; agreements; acts);

ii) search conducted in three databases [‘Science Direct’, 'Web of Science’, 'Scopus'] by using search terms such as: “effectiveness of decision making process Protected areas and Indigenous communities”; “women as decision makers in community forest management”:

iii) search conducted in ‘Science Direct’ and 'Web of Science' with search terms such as: “Amazon conservation policies”; “carbon market and local governance”; “overlapped areas between protected areas and indigenous territories Amazon”; "'Amazonia and deforestation and policies"; "historic trends" and "Amazonia".

iv) invited experts were asked to submit a written document to present a concrete example of the inclusion of diverse values of nature in a decision-making process at the local level.

.

**Process to screen the information gathered:**

Documents were screened based on their abstract (when academic papers) or their introductory paragraphs (when grey literature) and selected based on their relevance.

**Unit of the analysis:**

Documents (including on policy instruments, policy reports, academic papers, and case studies)

**N(initial - gathered):**

248 documents

**N(final - analysed):**

147 documents

**How was the evidence organised:**

Two spreadsheets were created to extract information on the following topics (and subtopics): i) decision-making (expression of values in decision-making, types of decision-makers, types of decisions, types of interactions among actors, decision outcomes and their context), and ii) selected Sustainable Development Goals (associated socioeconomic conditions, relations to nature’s contributions to people and nature).

As for the contribution received from external experts, the information was organised in a text document, by using a format that allowed to gather information on the context of the case and a description of a valuation process carried in the described context (which included the presentation of specific achievements and challenges faced during the process).

**Description of the analysis of the evidence:**

i) Valuation approaches and value types were identified focusing on the following information sources and strategies:

● Policy instruments: legislations; judicial decisions; chapters, articles and laws of two national Constitutions; court rulings; declarations; agreements; acts. Those policy instruments were analysed by applying content analysis. The latter entails, inter alia, a search of all forms, expressions or phrases related to the terms “value”, “valuation”, “importance”, “benefit of”, “meaning of”, “utility”, “origin”, “ancestral stewardship”, “creation”, “function”, “use of”, “sacredness of”, “nature”, “territory”, “ecosystems”, “protection principles”, “community vision”, “river”, “land”, “Mother Earth”, “harmony”, “equilibrium”, “biodiversity”, “biocultural rights”, “clean water”, “healthy environment”, “water as a fundamental right”, etc. Based on these terms and expressions, an inductive analysis was conducted moving from the specific to general information by categorizing information into different groups. Each cluster of information was thus organised into matrix tables whereby co-occurrence of value concepts and valuation relationships were easily identified.

● Scientific articles: design and use of review analysis tables containing columns with general themes such as the following: “References”, “[ILK & socio cultural] valuation methods”; “Theoretical approach - Guiding principles”; “Value types articulated”; “Nature's Contribution to People (NCP)”; “Purpose”; “Result/Outcomes”; “Spatial Scale - Location”; “Ways to achieve transformative change - Pathways for sustainability”; “Institutions”; “Power relations”; “Knowledge systems”; “Decision-making context”; “Critical assessment of the literature”. In this way, connections, contrasts, and similarities were more visible. Thus, a deductive analysis was performed moving from the general to specific information.

● Contributions from relevant stakeholders. The declaration of the Atrato river as subject of biocultural rights was a policy instrument identified. The analysis of the respective Constitutional court ruling (T-622)[22] enabled the identification of community councils and stakeholders with a key role in the leadership and outcome of the policy issue cycle. In this way, two of them were contacted and invited as contributing authors. A template for the case study was designed to collect concise first-hand information about the case and the valuation purpose in the policy issue cycle. A type of collaborative learning and knowledge exchange took place in the drafting of the case revolving around context, purpose and valuation methods, achievements and barriers in the policy issue cycle.

ii) Criteria for analysing decisions at local scales by applying the decision-making typology (DMT) of the IPBES values assessment[23]. The evidence was analysed according to the following phases:

● design and elaboration of spreadsheet,

● identification, selection and analysis of references (e.g., sociocultural and environmental literature; community webpages; interinstitutional agreements between Indigenous Authorities and National officers);

● grouping outcomes into categories based on stakeholders and process outcomes;

● assessment of decision impact across nature’s contributions to people and Sustainable Development Goals;

● analysis of relationships between stakeholders and network building.

iii) Forestry governance institutions with impact on the Amazon – A historic perspective. Historic trends in the expression of broad values were identified by analysing institutions (e.g., legislations, ordinances, forest codes, laws) in peer-reviewed articles, historic reports, special issues, international conferences, agreements, etc. Those sources were analysed by applying content analysis, which entails, inter alia, a search of all forms, expressions or phrases related to the terms “value” and “valuation”. Finally, a timeline of key governance institutions was elaborated with the respective values of nature conveyed through four historic periods (1530-1822, 1822-1900, 1900-2000, 2000-to now).

## **B.14. Review of valuation uptake into decisions**

**Table SI.B14:** Summary of review of valuation uptake into decisions

| **Specific topics supported:**  Valuation uptake in decision making | **Type of review:**  Semi-structured review partially based on expert criteria |
| --- | --- |
| **DOI of associated data management report:**  10.5281/zenodo.4391335 | **Type of sources:**  Peer-reviewed literature |
| **Section of the paper where this is covered:**  Section 2 | **Language(s) in which the review was carried:**  English, Spanish and French |

**Purpose of gathering the evidence:**

To identify the documented uptake of ecosystem (and ecosystem services) valuation cases in the peer reviewed scientific literature in relation to decision-making and policymaking at different levels and in different contexts and to identify causes for lacking uptake (identified as “blindspots”) and facilitators of best-practice uptake of valuation (identified as “brightspots”).

**Process followed to gather the evidence:**

A search on Web of Science was conducted covering scientific peer-reviewed publications between January 1981 and March 2020. Search terms were selected with the following constraints: i) keywords related to a valuation method family, ii) keywords related to the decision-making purpose (i.e., informative, decisive, technical) and iii) keywords related to nature and biodiversity. To be selected, a paper should contain a combination of words from all three constraints in their titles, abstracts or keyword lists.

**Process to screen the information gathered:**

The final database was obtained using random stratified sampling with strata of methods family (i.e., biophysical, integrating, multi-method, revealed preferences and stated preferences) and publication years (i.e., before 2000, 2000-2005, 2005-2010, 2010-2015, and after 2015) with the following 5 stages: i) an extensive search on Web of Science using a large set of keywords, 79,040 papers; ii) a stratified random sampling using methods family and publication year, 44,652 papers; iii) further stratified random sampling of methods family and publication year based on estimated reviewer capacity, 3,895 papers; iv) manual screening of sample in iii) by project leaders to reduce reviewing burden: 2,023 papers; v) review and coding of the final sample.

**Unit of the analysis:**

Papers

**N(initial - gathered):**

79,040 papers

**N(final - analysed):**

1,900 papers

**How was the evidence organised:**

Survey monkey was used by reviewers to enter data on each study, and then the database was transferred to Excel for graphics and STATA for analysis. The most important variables were study purpose (explorative, informative, decisive, technical), sub-purposes and levels of uptake (non, cursory, documented (testing/actual).

**Description of the analysis of the evidence:**

Out of the 1,900 coded documents, 178 were identified as documented uptake. The content of these papers was then validated to ensure all uptake papers in this category were indeed uptake cases. 37% of papers were confirmed to show evidence of uptake of valuation in decisions, which was used in quantifying the uncertainty in the coding. A lower bound of 3.5% and an upper bound of 15.7% was found to be documented uptake. A descriptive statistical analysis was conducted to describe general trends, means and relative averages.

## **B.15. Review of values associated with environmental certification outcomes**

**Table SI.B15:** Summary of review of values associated with environmental certification outcomes

| **Specific topics supported:**  Environmental certification outcomes | **Type of review:**  Semi-structured review partially based on expert criteria |
| --- | --- |
| **DOI of associated data management report:**  10.5281/zenodo.4394498 | **Type of sources:**  Peer reviewed literature and grey literature |
| **Section of the paper where this is covered:**  Section 3 | **Language(s) in which the review was carried:**  English |

**Purpose of gathering the evidence:**

To identify impacts (i.e., environment, livelihoods, institutional & social capital) of the on-the-ground implementation of sustainability certification; to assess values articulated by each actor in decision makings on sustainability adoption; and to compile and analyse the key findings and way forward from the adoption of sustainability certifications (within the ‘values’ they expressed and outcome/impacts they achieved)

**Process followed to gather the evidence:**

A search on Web of Science and Google Scholar was conducted covering scientific peer-reviewed publications between 1998 and 2020. Search terms were selected with the following constraints: i) keywords related to economically important agricultural commodities and seafood products, ii) keywords related to various sustainability standards and certifications and iii) keywords related to impacts on ecological and socioeconomics from the implementation of sustainability certification. To be selected, a paper needed to contain a combination of words from all three constraints in their titles, abstracts or keyword lists.

**Process to screen the information gathered:**

Evidence was filtered by searching reviews on sustainability certification schemes focused on commercially exported agricultural and seafood products through the search engine. Afterwards, other academic articles as well as reports were incorporated based on expert criteria. The final set was selected through expert judgement considering only papers reporting on outcomes emerging from the intervention (i.e. environmental certification).

**Unit of the analysis:**

Documents (academic papers and grey literature reports)

**N(initial - gathered):**

119 documents

**N(final - analysed):**

95 documents

**How was the evidence organised:**

A spreadsheet was created to add information coded from reviewing the full text of the selected documents. Retrieved information from the papers was related to the following topics: i) basic needs; ii) physical infrastructure; iii) fairness, social infrastructure; iv) ecological infrastructure; v) political economy; vi) institutional aspects (participation, politics, power, procedural justice, land tenure rights); vii) socio-cultural aspects (values, knowledge, crowding, social cohesion, recognition of justice); viii) livelihoods (income/poverty, equity, food security, well-being, distributive justice); and ix) environmental aspects (ecosystem services, effectiveness, efficiency, biodiversity; offsite/leakage)

**Description of the analysis of the evidence:**

Impact of certification schemes (ecological, economic or social) regarding specific commodities and types of certification schemes were analysed. An assessment was conducted on the agricultural and marine products commercially exported worldwide with significant ecological and socioeconomic impacts, such as oil palm, coffee, cacao, banana, soybean, timber, and seafood (not specified). The evidence was classified into different certification schemes applied and focused on quantitative data of impacts. The socioeconomic impacts of sustainability certification were focused on smallholders, as the most marginalised actors along the value chain.

## **B.16. Review of values and outcomes in protected areas and Indigenous community conserved areas**

**Table SI.B16:** Summary of review of values and outcomes in protected areas and Indigenous community conserved areas

| **Specific topics supported:**  Outcomes in protected areas and Indigenous and community conserved areas (ICCAs) in the context of diverse values | **Type of review:**  Combination of reviews:  ● Semi-structured review partially based on expert criteria  ● Non-structured review fully based on expert criteria  ● Invited contributions from external experts and stakeholders |
| --- | --- |
| **DOI of associated data management report:**  10.5281/zenodo.4394267 | **Type of sources:**  Peer reviewed literature, grey literature, contributions from summoned experts |
| **Section of the paper where this is covered:**  Section 3 | **Language(s) in which the review was carried:**  English, Polish |

**Purpose of gathering the evidence:**

To inventory the types of outcomes measured for protected areas, and to link different causal mechanisms to different outcomes (for the impact evaluation with robust counterfactuals only). This required assessing case studies for national protected areas (NPAs) to describe the values, knowledge, and processes (including power relations) involved in establishment and management of the protected area, and evaluate the social, economic, and ecological outcomes of the protected area. This evidence was used to provide more insight and nuance into the context in which certain outcomes were seen. Case studies for Indigenous and Community Conserved Areas (ICCAS) were considered to: i) assess the values and rules that guide resource management; ii) examine the legal and environmental implications of customary governance; iii) compile the key findings within each case study and determine the way that decision-making is articulated in each case

**Process followed to gather the evidence:**

● Review of reviews. A search was conducted in Web of Science using the following terms: (TI=("protected area*" AND (outcome* OR impact* OR effect* OR conflict* OR poverty* OR social))) AND DOCUMENT TYPES: (Review) Total results: 50 records. - Other studies were then mined from those reviews.

● Case studies – NPAs. Specific PAs with the most evidence on outcomes in the peer reviewed literature were identified from the review of reviews, and authors of those studies were contacted and asked to contribute. Those contributing authors conducted their own reviews or drew from their expert knowledge of the evidence regarding those PAs.

● Case studies – ICCAS. A search was conducted in Web of Science and Google Scholar. Terms used were: "Indigenous" and/or "cultural" and "community conserved areas" OR "cultural landscapes" and/or "governance". Total results: 54 records.

**Process to screen the information gathered:**

● Review of reviews. Abstracts were screened for relevance, if titles of studies included in review were screened for relevance (either documenting actual outcomes in a specific PA or set of PAs, or reviewing other studies that did)

● Case studies – NPAs. Only those studies related to the specific PA being reviewed were considered, but it was up to expert discretion whether the study provided useful context to understanding the conditions under which the outcomes in that PA occurred.

● Case studies – ICCAS. Papers were scanned on title, reference to the term 'indigenous' or 'cultural' and selected based on regional diversity (one from each continent). Expert analysis was used to select the sites and offer multiple viewpoints reflective of geographic variation.

**Unit of the analysis:**

For individual protected areas, the unit of analyses are study-sites (described in papers, and protected areas contained within them). It is important to distinguishing from the number of papers because some papers contained multiple sites that were evaluated) and the number of total outcomes reported. For impact evaluation reviews, the unit of analysis were the number of studies reviewed or the number of protected areas included in the analysis and the number of total study-outcome units reported (which were far fewer than the total number of studies or number of protected areas comprising the impact evaluation reviews, because hundreds of studies could all be examined for a single outcome).

**N(initial - gathered):**

● 1,202 site-study units for individual protected areas

● 120 Impact evaluation studies (within 96 papers)

● >200 studies across 11 case studies (on NPAs and ICCAS)

**N(final - analysed):**

● 482 site-study units reporting on 720 outcome units at individual protected areas

● 55 Impact Evaluation studies (reviewing collectively >12,000 protected areas) for a total of 68 outcome units

● 11 case studies (7 on NPAs, 4 on ICCAs)

**How was the evidence organised:**

● Individual PAs were organised by protected area and by country, and the following fields were tracked: impact (or outcome) and method for measuring it. All were classified according to the IPBES conceptual framework, including the following: nature, nature’s contributions to people, good quality of life, anthropogenic assets, institutions, drivers, values (change), conflicts.

● Impact evaluation studies (for multiple PAs) were organised by study and by country (or region, or global), and the following fields were tracked: impact (or outcome), method for measuring it, mechanisms proposed determining it (if stated), and directionality of the outcome. All impacts/outcomes were classified according to the IPBES conceptual framework, including the following: nature, nature’s contributions to people, good quality of life, and other.

● ICCAS review: key terms were coded intext files and spreadsheets; terms included: Indigenous, region, ICCA, CCA, cultural landscape.

**Description of the analysis of the evidence**

● Studies identified through reviews were tallied by number of studies in different geographies and number of different measures for the various outcomes categories (nature, NCP, good quality of life). This was basically a descriptive exercise to see what types of outcomes were being measured, how diverse values were represented in those measurements.

● Impact evaluation studies were analysed using the following hypotheses: greater degrees of local participation or autonomy in establishment and management of protected areas are associated with better outcomes; protected areas established with multiple use (indicating multiple values) as a goal exhibit a greater degree of win-wins for social and ecological outcomes.

● Case studies were analysed with the following contextual questions: i) Brief outline of the PA/ICCA/Indigenous landscape: where is it?; ii) Brief outline of the socio-economic conditions: historical and/or current land tenure or legal issues? inequities? Legacies of colonisation? disenfranchisement?; iii) Brief outline of any political conditions/governance context (relevant institutional framework and policy; Indigenous territoriality?); iv) Goals: What were the goals of the reserve? What is the designation? What are the trade-offs? What are the levels of protection?; v) Values: What values guide protection? Whose values were reflected/excluded? Does it contribute to language and/or cultural revitalization? How does the PA represent and support Indigenous/local peoples’ rights?; vi) Enforcement: How were these restrictions enforced? Who guides access and how? Who makes the decisions regarding changes/threats/new ideas?; vii) Economics: Is there a plan for financial sustainability? Does it enhance aspirations for sustainable community economic development?; viii) What have been the challenges and opportunities to date? How has the protection status changed over time, or any management changes altered?; ix) What outcomes have been evaluated? Were they positive or negative? What were the trade-offs?

## **B.17. Review of values and outcomes of Payments for Ecosystem Services**

**Table SI.B17:** Summary of review of values and outcomes of Payments for Ecosystem Services

| **Specific topics supported:**  Payments for ecosystem services and their outcomes under diverse values | **Type of review:**  Combination of reviews:  ● Comprehensive structured review  ● Semi-structured review partially based on expert criteria  ● Non-structured review fully based on expert criteria  ● Invited contributions from external experts and stakeholders |
| --- | --- |
| **DOI of associated data management report:**  10.5281/zenodo.4394520 | **Type of sources:**  Peer reviewed literature, grey literature and contributions written by invited external experts |
| **Section of the paper where this is covered:**  Section 3 | **Language(s) in which the review was carried:**  English, Spanish |

**Purpose of gathering the evidence:**

To determine i) what factors have been shown to influence payments for ecosystem services (PES) outcomes and by what mechanisms; and ii) how these factors relate to (mis)alignments among local and program values, institutions, and knowledge systems, in order to iii) assess the potential for plural valuation to enhance PES outcomes. This review was completed with deep case studies involving reviews based on expert criteria that further examine how misalignments arise in PES and how they influence outcomes, and to identify iv) what value articulation processes are most effective at aligning local values, institutions, and knowledge systems with PES design and implementation; and v) what contextual factors support/undermine these efforts.

**Process followed to gather the evidence:**

Keyword search in academic datasets for review papers. The terms used for the search were:

payment* for ecosystem services" OR "payment* for watershed services" OR "investment* in watershed services" OR "water fund*" OR "payment* for environmental services" OR "investment* in environmental services" OR "investment* in ecosystem services" OR "investment* in ecological services" OR "payment* for ecological services" OR REDD OR REDD+) AND TS = ("outcome*" OR "impact*" OR "leakage" OR "biodiversity" OR “justice” OR “equity”) Refined by: "review

Then, based on a set of criteria regarding availability of information and representation of different regions, governance structures and scales, and biophysical contexts, eight case studies were chosen and experts were asked to provide an in-depth assessment on the literature for these cases.

**Process to screen the information gathered:**

Review papers were screened based on title and abstract and tagged according to themes of relevance and geographical scope, with the most relevant papers given a “starred” tag, which was then used to prioritise the papers. This prioritisation yielded 40 starred review papers of highest priority which covered impacts of programs in more than one country. As for the case studies, all eight case studies were considered.

**Unit of the analysis:**

For the main literature review, papers (rather than case studies covered in each paper) were the unit of analysis. For the case-study part of the review, the unit were the cases themselves.

**N(initial - gathered):**

275 papers and 8 case studies

**N(final - analysed):**

40 papers and 8 case studies

**How was the evidence organised:**

For the main literature review a spreadsheet was used and the main fields include: factors influencing outcomes; direction and mechanism of influence; outcomes affected; fFeedback effects (onto other outcomes/factors). As for the case studies, these were organised within the individual text documents that the contributors created.

**Description of the analysis of the evidence:**

For the main review, the framework from Bayrak and Marafa (2016)[24] was adapted to categorise impacts in five dimensions: institutional, livelihood, socio-cultural, target ecosystem services, and other environmental outcomes. An emergent coding approach was used to identify influencing factors, the mechanisms by which they influence various outcomes, and the feedbacks among them. These factors were grouped into broad categories, identifying which ones were related to the (mis)alignment between PES design/implementation and participant values, knowledge systems, and institutions.

For the case studies, a 3-point scale was used to score each program according to how effectively it addressed multiple dimensions of justice at the problematization, design, and implementation stages. These scores provided a tool to track how (mis)alignments among values, institutions, and knowledge systems relate to multiple dimensions of justice, where these (mis)alignments occur, how they affect program sustainability and outcomes, and how they may evolve within a given program over time.

The focus on (mis)alignments differs from other reviews that have correlated various design or contextual features with PES success (Calvet-Mir et al. 2015[25]; Boerner et al. 2017[26]) by highlighting the interactions among program design and contextual elements as well as the mechanisms by which these influence outcomes.

## **B.18. Review of values articulated in major infrastructure and development projects**

**Table SI.B18:** Summary of review of values articulated in major infrastructure and development projects

| **Specific topics supported:**  Value articulation in the context of major infrastructure and development projects | **Type of review:**  Invited contributions from external experts and stakeholders |
| --- | --- |
| **DOI of associated data management report:**  10.5281/zenodo.4395985 | **Type of sources:**  Peer-reviewed literature and grey literature |
| **Section of the paper where this is covered:**  Section 3 | **Language(s) in which the review was carried:**  English & Spanish |

**Purpose of gathering the evidence:**

To understand how values towards nature and other values are articulated and represented, and what other knowledge and worldviews go into the decision-making, around big (largely irreversible projects) infrastructure and development projects such as dams and mines.

**Process followed to gather the evidence:**

Google Scholar and expert consultation was used to identify review papers on the final outcomes of mining and big dam projects, including the mega-review on which the Report of the World Commission on Dams was based. For relating outcomes to decision-making processes and the values and knowledges mobilized in them, a case study approach was used. Case studies were provided by invited authors who used their experience, expertise and past writings to compile their contributions. Special efforts were made to ensure broad geographical coverage (South Asia, Middle East, South America, North America). Two main 'project' types were the focus: mining and big dams. Each case study covered the following questions: what kinds of values and extent of benefits under them were highlighted in the proposal?; what kinds of negative impacts were originally highlighted in the proposal?; what measures were proposed to mitigate these losses/impacts?; what methods were used to assess the impacts and balance them against benefits?; what methods were officially to be used in decision making?; what values were foregrounded in the opposition to the project?; what forms of knowledge were foregrounded?

**Process to screen the information gathered:**

Only those projects which caused (or could have caused) major ecosystem transformation/disruption, and for which more than 10 peer-reviewed papers were associated with and available for review, for which specifically analysis of the values articulated in decision-making was available, were considered for inviting case study write-ups from experts.

**Unit of the analysis:**

Case studies centred around specific major mining or dam projects, and one consultation with an expert on their experience spanning many dam projects across multiple continents.

**N(initial - gathered):**

5 case studies and 1 multi-project consultation.

**N(final - analysed):**

5 case studies and 1 multi-project consultation.

**How was the evidence organised:**

Each case study contribution was kept in the separate text documents created by the experts who provided them. The multi-project consultation was converted into a note authored by the expert in response to the same questions as posed in individual case studies.

**Description of the analysis of the evidence:**

The review papers were used to evaluate outcomes of big dam and mining projects in general in terms of 3 dimensions: immediate human well-being (including material/instrumental and intrinsic values), sustainability (of the well-being provided), and justice (distribution of the well-being gains and losses geography and social position).

The analysis of the case studies focused on the relationship between outcomes and i) the kinds of values and knowledges that were mobilised by proponents and opponents of the projects at various stages of decision-making, and ii) the nature of formal decision-making processes and their consequences. Specifically, the values mobilized were categorized into instrumental values (associated with material well-being), relational values towards nature, intrinsic value of nature, and also inter-personal values, viz., intra-generational equity and inter-generational equity or sustainability, and values for process, i.e., recognition and procedural justice. Knowledge mobilized was categorized into modern scientific knowledge and traditional/local knowledge. Decision-making processes were divided into two stages—initial officially mandated processes and then changes brought about due to protest and conflict, if any. The official processes were assessed using 4 key principles of the ‘gold standard’ articulated by the World Commission on Dams (recognition of rights of affected communities, inclusion of stakeholders in decision-making, free-prior-informed-consent (FPIC) from indigenous communities, and transparent processes) which are also shared by the Extractive Industries Transparency Initiative for mining projects. The changed processes were categorized into through brought about through national and international protest, those brought about by approaching the national judiciary, and those brought about by street protest.

## **B.19. Review of power dynamics in philosophies of good living**

**Table SI.B19:** Summary of review of power dynamics in philosophies of good living

| **Specific topics supported:**  Power dynamics in “good living” philosophies | **Type of review:**  Comprehensive structured review |
| --- | --- |
| **DOI of associated data management report:**  10.5281/zenodo.4399544 | **Type of sources:**  Peer-reviewed literature |
| **Section of the paper where this is covered:**  Sections: 2, 5 | **Language(s) in which the review was carried:**  English, Spanish, French |

**Purpose of gathering the evidence:**

To gather evidence regarding the links between power relations and diverse philosophies of good living.

**Process followed to gather the evidence:**

Two searches were conducted in Scopus and Web of science using the following search terms.

● Search 1: TS= (TITLE-ABS-KEY ("buen vivir" OR "vivir bien" OR "living well" OR "good living" OR “collective wellbeing” OR “well living” OR “Mauri Ora” OR “good life” OR “Alli Kawsay” OR “Laman Laka” OR “Minobimaatisiiwin” OR “pura vida” OR “vida sabrosa” OR “comunalidad” OR “Sumak Kawsay” OR “harmonious life” OR “Suma Qamaña” OR “Kiwe & Sek Taki” OR “Ubuntu” OR “Unhu” OR “Umunthu” OR “Satoyama” OR “satoumi” OR “Kuleana” OR ”Úxwalmixwts” OR “lekil kuxlejal” OR “teko pora”) AND TITLE-ABS-KEY (valu* OR worldview* OR cosmovision* OR perspective* OR philosoph* OR “traditional knowledge” OR “traditional ecological knowledge” OR “knowledge” OR “local knowledge” OR “indigenous frameworks” OR “kinship” OR “principles”) AND TITLE-ABS-KEY (indigen* OR tribes OR aborigin* OR autochthonous OR pastoralis* OR herder OR fisher* OR hunt* OR forest* OR agriculture OR livelihood OR nomad OR afro descendant OR afro-descendant OR raizales OR mestizo OR campesin* OR peasant OR creol* OR kriol OR urban)

● Search 2:TS= (TITLE-ABS-KEY ("buen vivir" OR "vivir bien" OR "living well" OR "good living" OR “collective wellbeing” OR “well living” OR “Mauri Ora” OR “good life” OR “Alli Kawsay” OR “Laman Laka” OR “Minobimaatisiiwin” OR “pura vida” OR “vida sabrosa” OR “comunalidad” OR “Sumak Kawsay” OR “harmonious life” OR “Suma Qamaña” OR “Kiwe & Sek Taki” OR “Ubuntu” OR “Unhu” OR “Umunthu” OR “Satoyama” OR “satoumi” OR “Kuleana” OR ”Úxwalmixwts” OR “lekil kuxlejal”) AND TITLE-ABS-KEY (valu* OR worldview* OR cosmovision* OR perspective* OR philosoph* OR “traditional knowledge” OR “traditional ecological knowledge” OR “knowledge” OR “local knowledge” OR “indigenous frameworks” OR “kinship” OR “principles”) AND TITLE-ABS-KEY (indigen* OR tribes OR aborigin* OR autochthonous OR Pastoralis* OR herder OR fisher* OR hunt* OR forest* OR agriculture OR livelihood OR nomad)

**Process to screen the information gathered:**

Abstracts and titles were screened. Relevant documents were retrieved and coded (215 papers). One code provided information on power and decision-making (140 papers) .

**Unit of the analysis:**

Papers

**N(initial - gathered):**

215 papers

**N(final - analysed):**

140 papers

**How was the evidence organised:**

Spreadsheet with 24 codes grouped in the following general topics: i) information about the document; ii) relation with Indigenous and local knowledge values; iii) excerpts supporting predefined Ingigenous and Local Knowledge (ILK) topics; and iv) power.

**Description of the analysis of the evidence:**

Implicit and explicit information on the general topic of “power” (number iv above) was identified. Specific excerpts from the papers were categorised based on the specific aspect related to “power” that they covered, such as: i) decoloniality & education; ii) challenge the system - decolonizing methods; iii) distributive justice; iv) dispossession; and v) decision-making. Using these excerpts and categories, information was qualitatively interpreted and summarized to provide understanding on the relationships between values in good living philosophies, power and decision-making.

## **B.20. Review of values in agrobiodiversity conservation**

**Table SI.B20:** Summary of review of values in agrobiodiversity conservation

| **Specific topics supported:**  Values articulated in the context of agrobiodiversity management | **Type of review:**  Combination of reviews:  ● Comprehensive structured review  ● Semi-structured review partially based on expert criteria |
| --- | --- |
| **DOI of associated data management report:**  10.5281/zenodo.4394547 | **Type of sources:**  Peer-reviewed literature |
| **Section of the paper where this is covered:**  Section 3 | **Language(s) in which the review was carried:**  English |

**Purpose of gathering the evidence:**

To identify the values of crop diversity for farmers and to understand how these values are involved in decisions related to agriculture

**Process followed to gather the evidence:**

A search was conducted in the Web of Science, considering publications from all years and using search terms related to farmers, values and agriculture.

**Process to screen the information gathered:**

Documents included had to meet the following criteria: i) studies based on primary sources; ii) studies based on crops actually grown by farmers and iii) studies focusing on farmers point of view. The final selection of papers considered the representation of diverse countries and cropping systems. The sample obtained from Web of Science was completed with studies known by the experts who conducted the review.

**Unit of the analysis:**

Papers

**N(initial - gathered):**

3,719 papers

**N(final - analysed):**

12 papers (each one associated with a different case study)

**How was the evidence organised:**

Values were coded for each paper in spreadsheets focusing on the following aspects: i) The description of the agroecosystem, ii) the agroecosystem’s trend, iii) the scale of the farming system, iv) the main objective of the farmers in the study, v) the values identified (in general), vi) the value types identified (specifically), vii) valuation methods, viii) if there were synergies or tradeoffs mentioned and their description, ix) documented outcomes from agrobiodiversity management, x) mentions to policy instruments, xi) outcomes from the policy instruments in terms of encouraging associated biodiversity, and xii) outcomes from the policy instruments in terms of encouraging crop diversity.

**Description of the analysis of the evidence:**

As the articles did not systematically use the concept of value, proxies indicating values (what people care for) and the associated valuations (the process of developing values through experience and choices made), were looked for. The proxies were a set of indicators that allowed us to assume the existence of values and correspond to the search terms. These proxies correspond to various registers of valuation (practical, rational or emotional), including preference, decision, choice, motivation, justification, attachment, utilisation, judgement, consideration, effective valuation, care, perception, point of view, action, management, concern, and rationale. Valuations are the elements or qualities sought or appreciated by farmers. Based on a set of 12 case studies selected from the literature, these proxies were extracted and then, organised thematically in a tree of valuation processes. Information about the drivers shaping local decision-making regarding crop diversity and related governance challenges was also obtained.

## **B.21. Review of values articulated by Indigenous peoples and local community (IPLC) institutions**

**Table SI.B21:** Summary of review values articulated by Indigenous peoples and local community (IPLC) institutions

| **Specific topics supported:**  Values revealed by Indigenous and local community institutions | **Type of review:**  Combination of reviews:  ● Invited contributions from external experts and stakeholders  ● Comprehensive structured review  ● Non-structured review fully based on expert criteria  ● Semi-structured review partially based on expert criteria |
| --- | --- |
| **DOI of associated data management report:**  N/A | **Type of sources:**  Peer-reviewed literature |
| **Section of the paper where this is covered:**  Sections 1, 2, 3, 4, 5, 6 | **Language(s) in which the review was carried:**  English, Spanish, French |

**Purpose of gathering the evidence:**

To assess evidence regarding values revealed by indigenous peoples and local communities (IPLC) institutions

**Process followed to gather the evidence:**

● Review of reviews. Search was conducted in Web of Science. The search terms applied were: (TI=("protected area*" AND (outcome* OR impact* OR effect* OR conflict* OR poverty* OR social))) AND DOCUMENT TYPES: (Review). Total results: 50. Other studies were then mined from those reviews.

● Case studies, natural protected areas: specific protected areas with the most evidence on outcomes in the peer reviewed literature were identified from a review of reviews, and authors of those studies were contacted and asked to contribute. Those contributing authors conducted their own reviews or drew from their expert knowledge of the evidence regarding those protected areas.

● Case studies of indigenous community conserved areas. Search conducted in Web of Science and Google Scholar. Terms used were: "Indigenous" and/or "cultural" and "community conserved areas" OR "cultural landscapes" and/or "governance". Total results: 54.

**Process to screen the information gathered:**

Based on expert criteria, relevant documents were selected.

**Unit of the analysis:**

Documents (scientific papers, case studies, contributions from Indigenous and Local Knowledge (ILK) holders and experts )

**N(initial - gathered):**

147

**N(final - analysed):**

51

**How was the evidence organised:**

Spreadsheet created to code the chosen documents. Excel spreadsheet was used to code and track themes around “Indigenous" and/or "cultural" and "community conserved areas" OR "cultural landscapes" and/or "governance". From the total 54 results, these keywords were interpreted for context and interpreted for ideas found within the local understanding or ethnolinguistic interpretation. Important quotes were flagged, and comments were added to capture emerging themes and notes. Information gleaned from the Contributors on ICCAs Case Studies and the Literature review for the chapter added to the evidence. The common themes were then placed in a word document for analysis and sources to obtain a large geographical net of information for comparison and text.

**Description of the analysis of the evidence:**

The mentions to philosophies of good living and territorial rights were identified and extracted from the literature. The coded articles were flagged for ethnolingual words such as ‘mino bimaadiziwin’, ‘good life’, ‘dobrobyt’, or ‘balanced life’, to provide a meta-analysis of the connection between the philosophy and territorial governance. The arguments were gathered, and a rough outline was created for common and contradicting themes. The analysis was contextualised to show how different cultural groups rely on the philosophy of good living’ to formulate their knowledge of the environment and governance. The flagging of the different processes that cultural groups formulate their ‘good life’ illustrates the interrelatedness of all components of the natural environment, respectful attitudes towards each member of society (human and non), and well as a standard of life that meets local requirements. Expressed in different ways, these components together have been identified in the research analysis as foundational elements of the coded theme of ‘territoriality’. The meta-analysis was peer-reviewed by the experts of the values assessment.

## **B.22. Review of valuation and Aichi Target 2 reporting**

**Table SI.B22:** Summary of review of valuation and Aichi Target 2 reporting

| **Specific topics supported:**  Valuation of biodiversity and Aichi target 2 reporting at country level | **Type of review:**  Semi-structured review partially based on expert criteria |
| --- | --- |
| **DOI of associated data management report:**  10.5281/zenodo.6468917 | **Type of sources:**  Peer reviewed literature and grey literature |
| **Section of the paper where this is covered:**  Section 2 | **Language(s) in which the review was carried:**  English |

**Purpose of gathering the evidence:**

To explore the correlation between the number of valuation studies conducted in a country, the level of implementation of SEEA EA[27] (ecosystem accounting) and the level of reporting of valuation of biodiversity and accounting to the CBD[28] by National Report on progress relative to Aichi target #2.

**Process followed to gather the evidence:**

There were three processes for the review, each with its own starting and ending point: i) from the corpus obtained for a separate review[29] (See previous document number 11 on “valuation methods and approaches”) the number of valuation studies per country was obtained; ii) reports on progress on Aichi target 2 were obtained for each of the analyzed countries (updated until January 2021, courtesy of the CBD Secretariat); and iii) details regarding information on the implementation of SEEA EA implementation were consulted in the SEEA EA website[30].

**Process to screen the information gathered:**

No filtering was performed; all data obtained was considered.

**Unit of the analysis:**

● Mixed unit of analysis including: Published papers, SEEA EA implementation initiatives; National Reports of progress towards Aichi #2

**N(initial - gathered):**

Data sources:

● From the separate literature review[31], N=48,781 papers with a georeference in Web of Science referring to data for 217 countries.

● Aichi #2 in National Biodiversity Strategy Action Plans as of Jan. 2021 from CBD Secretariat N=193 entries at country level (countries under the category “unknown” are not considered in these numbers).

● SEEA EA 2020 Global Assessment Results N=116 entries at country level.

**N(final - analysed):**

All data was included for the analysis, a total of 249 Countries were considered composed by countries mentioned in either one of the three data sources above.

**How was the evidence organised:**

Spreadsheet in a csv file was imported to Hugin Expert statistical software. The main variables and their categories for the valuation atlas were

● the number of valuation studies per country categorized

o very limited valuation research (<100 studies)

o limited (100-200 studies)

o some (200-1000 studies);

o high (>1000 studies)

● For Aichi #2 the progress towards the target was assessed

o 24.4% = “target exceeded” (0,73%) +

o 23.64% “on track to reach”

o 31% “some progress”

o 3% “no change”

o 0.4% “moving away from target”

o 41% = “not reported” and “unknown”

● For SEEA data, the degree of implementation was considered

o Implements SEEA and was confirmed by international agencies and UN regional commissions

o Implementation data confirmed by international agencies and UN regional commissions

o Implements SEEA

o Planning to implement SEEA

**Description of the analysis of the evidence:**

Correlations between “number of valuation studies”, “progress towards Aichi target 2” and “state of incorporation of UN SEEA EA” were visualised in a Sanky diagram. Hypotheses about causality between variables were assessed using a Bayesian network. The Bayesian network allowed to carry out inference and diagnostics, answering questions such as given that a country reports "insufficient progress" in achieving Aichi target 2, what is the likelihood that ecosystem accounting has been applied in the country and the country has a high implementation of valuation studies; or conversely given that SEEA EA has been implemented in the country, what is the likelihood that National Reports to CBD are rated as achieving Aichi target #2.

## **B.23. Review on values and future scenarios**

**Table SI.B23:** Summary of review on values and future scenarios

| **Specific topics supported:**  The diversity of values of nature that underpin multiple pathways towards sustainability | **Type of review:**  Semi-structured review partially based on expert criteria |
| --- | --- |
| **DOI of associated data management report:**  10.5281/zenodo.4359655 | **Type of sources:**  Peer-reviewed literature and grey literature |
| **Section of the paper where this is covered:**  Section 5 | **Language(s) in which the review was carried:**  English, Japanese |

**Purpose of gathering the evidence:**

To assess the values underlying portrayals of the future and the outcomes relating to nature, nature's contributions to people and good quality of life.

**Process followed to gather the evidence:**

A combination of keywords was applied to identify relevant peer-reviewed literature in Web of Science and grey literature in Google Custom Search Engines. Then, a selection of documents based on expert criteria and a snowballing approach was performed.

**Process to screen the information gathered:**

Iterative process based on research area, title and abstracts and by expert criteria were used to assess whether it contained scenarios addressing values of nature, nature's contributions to people and good quality of life.

**Unit of the analysis:**

Scenario (within relevant papers)

**N(initial - gathered):**

1,205 scenarios found across all papers identified as relevant

**N(final - analysed):**

460 scenarios analysed across peer-reviewed papers and grey-literature

**How was the evidence organised:**

The final selection of papers was organised in a database with n=45 fields, with the aim to capture the relationship between the values underpinning the development in each scenario, their interaction with other driving forces, and the resulting potential future impact on nature, nature’s contributions to people and good quality of life. The five broad categories of fields were as follows: scenario description (type, origin, timeframe, etc.), scenario development (stakeholder involvement, ILK inclusion, etc.), policy & management & actions (specific response options included in the scenarios), drivers and outcomes (drivers of the scenario dynamics and their impacts on nature, NCPs and good quality of life), values included in the scenarios (types of values and valuation implicitly or explicitly captured in the scenario).

**Description of the analysis of the evidence:**

Qualitative, semi-quantitative and quantitative approaches were used to i) assess the similarity of the scenarios in the database, ii) categorise the scenarios into scenario archetypes, and iii) assess the types of values underpinning each broad scenario archetype. The similarity of the scenarios was assessed first qualitatively based on the text description of drivers and outcomes as coded in the database. The similarity between the scenarios was assessed using text analysis (similarity matrices). Similar types of scenarios were grouped into scenario archetypes, as defined by the IPBES Scenarios and Models assessment[32]. Finally, for each archetype, the frequency of different types of values underpinning potential future development was calculated.

## **B.24. Review of the role of values in transformative change**

**Table SI.B24:** Summary of review of the role of values in transformative change

| **Specific topics supported:**  Role of values in transformative change | **Type of review:**  Semi-structured review partially based on expert criteria |
| --- | --- |
| **DOI of associated data management report:**  10.5281/zenodo.4363069 | **Type of sources:**  Peer reviewed literature |
| **Section of the paper where this is covered:**  Sections 1, 4, 6, 7 | **Language(s) in which the review was carried:**  English |

**Purpose of gathering the evidence:**

To understand the role of values in transformations towards preferred futures and explore: i) how can plural values of nature contribute to transformations towards just and sustainable futures; ii) what constrains and enables the mobilisation of plural values of nature for transformations to sustainability?; and iii) how can the mobilisation of plural values of nature be governed for transformations to sustainability?

**Process followed to gather the evidence:**

A search guided by keywords was conducted in Web of Science. Keywords used were:

TI=(value* or valuation or ethic* or moral* or belief) AND TS=(transformat* or transition*) AND TS=(sustainab* or adaptation).

Additionally, high relevance papers were selected through expert knowledge.

**Process to screen the information gathered:**

Titles and abstracts were screened and based on expert criteria, papers that clearly presented information on transformations or transitions to sustainability, and which included an environmental and social dimension, to address individual or social values, were selected.

**Unit of the analysis:**

Papers

**N(initial - gathered):**

437 papers

**N(final - analysed):**

149 papers

**How was the evidence organised:**

A spreadsheet was created. The main fields used were: theories and conceptualizations on the role of values in transformative change towards just and sustainable futures; role of values in transformative individual behaviour change and how interventions can promote change for biodiversity conservation ; values as leverage points for transformational change and what interventions can help to mobilise these levers.

**Description of the analysis of the evidence:**

● Qualitative content analysis of selected literature confirming expert knowledge of well established body of psychology work on the 'gap' between values and behaviour

● Qualitative analysis + semi-quantitative: During review, coders identified whether papers discussed particular values or value types that belonged to a sustainable and just future and were asked to note them.

● Qualitative analysis: During review, 13 conceptual frameworks contained in the literature were reviewed, finding strong agreement for the idea of combined operation across different places of intervention (leverage points).

● Qualitative analysis: The review elaborated on the results of IPBES global assessment[33] finding that transformative change is 'system-wide'. The weight of evidence in the reviewed papers was found system-wide to incorporate multiple societal spheres of practice (technology, economy, society) as well as underlying paradigms and goals.

● Qualitative analysis + semi-quantitative: Particular analysis of literature on leverage points (a subset of database) found the idea of 'deep' leverage points to be well established.

● Semi-quantitative: The role of power was a predefined category/column heading in the database. Analysis of this data column found that the function of power asymmetries as a barrier to transition and transformational change was well established.

● Qualitative analysis + semi-quantitative: Particular analysis of literature on leverage points (a sub-set of database) found the idea of 'deep' leverage points to be well established. Societal goals and norms were most frequently cited as deep leverage points.

## **B.25. Review of values associated with pathways to sustainability**

**Table SI.B25:** Summary of review of values associated with pathways to sustainability

| **Specific topics supported:**  Broad and specific values associated with pathways to sustainability | **Type of review:**  Non-structured review fully based on expert criteria |
| --- | --- |
| **DOI of associated data management report:**  N/A | **Type of sources:**  Peer-reviewed papers  Grey literature |
| **Section of the paper where this is covered:**  Section 5 | **Language(s) in which the review was carried:**  English |

**Purpose of gathering the evidence:**

To explore how different pathways to sustainability are founded on (and refer to) broad and specific values

**Process followed to gather the evidence:**

A set of pathways that represents the diversity of prioritised specific values (instrumental, intrinsic and relational) was chosen. For a pathway to be selected, the following criteria had to be met:

● it had a recognizable coalition of advocates with particular knowledge resources and institutions, including dedicated theory, textbooks, journals, and conferences;

● it was very widely known, well-developed, with a track record of significant or growing momentum, not only in academia but also in policy debates;

● it was global in its relevance and in research contributions, even if it may have origins in specific regional academic traditions.

Key literature for each of the four selected pathways was identified through expert based knowledge.

**Process to screen the information gathered:**

All information selected for each pathway was reviewed

**Unit of the analysis:**

Case study pathway

**N(initial - gathered):**

150

**N(final - analysed):**

150

**How was the evidence organised:**

Six questions were used to structure evidence from source materials: i) what are the key bodies of theory/knowledge that underlie pathways for sustainability?; ii) what does the pathway identify as the key driver(s) of biodiversity loss?; iii) what does the pathway identify as key requirements of transformation towards sustainability?; iv) what values (human-human and human-nature) are highlighted as aligned with sustainability?; v) what is the core values agenda (e.g. making values visible, ensuring uptake in decision-making, etc); vi) what are the emblematic policy solutions proposed by advocates of this pathway?

**Description of the analysis of the evidence:**

Literature for each pathway was summarised based on the six guiding questions. A subsequent analytical stage collated and further summarised these pathway reviews into tabular format. Comparative analysis then identified key differences and commonalities.

## **B.26. Review of values-centred policy-making options for transformative change**

**Table SI.B26:** Summary of review of values-centred policy-making options for transformative change

| **Specific topics supported:**  Options for policymaking to engage with the diversity of values for transformative change | **Type of review:**  Combination of reviews:  ● Invited contributions from external experts and stakeholders  ● Non-structured review fully based on expert criteria. |
| --- | --- |
| **DOI of associated data management report:**  N/A | **Type of sources:**  Peer-reviewed literature, grey literature and contributions from summoned experts, other (chapters 1 - 6 of the IPBES values assessment) |
| **Section of the paper where this is covered:**  Sections 4, 6 | **Language(s) in which the review was carried:**  English |

**Purpose of gathering the evidence:**

To identify existing sectoral and cross-sectoral policy options to operationalize the diverse values of nature in decision-making and to provide guidelines to better incorporate the diverse values of nature in policy decisions.

**Process followed to gather the evidence:**

To fulfil the first purpose, multiple experts were summoned to provide information on how policy options within and across sectors are implemented. The review started with the identification, based on expert criteria, of relevant cross-sectoral policy options. Keywords that refer to the different policy sectors and cross-sectoral themes were selected. Multiple targeted literature reviews were carried by each of the summoned experts, in databases such as Web of Knowledge, ScienceDirect and Google Scholar. The information gathered was complemented by expert-based selected literature and case studies. Each of the reviews referred to a specific sector: urban transformation, landscape conversion (case study of nuclear waste management), agriculture, protected areas, health, education, and economy. For the second purpose, evidence related to the operationalization of values in decision making was gathered from chapters 1 - 6 of the IPBES values assessment and complemented with information selected from searches in scientific and other databases, as well as with evidence selected based on expert-based knowledge.

**Process to screen the information gathered:**

For the first purpose, each review had its own process to filter the evidence gathered, but in general, documents relating multiple values to different governance sectors or cross-sectors were selected, synthesised and used to portray case studies when it was necessary to provide examples. For the second purpose, the evidence was filtered based on expert criteria, by selecting those pieces of evidence that provided information on the operationalization of nature’s values across different contexts and stakeholders. The information identified was selected and classified in a set of operationalization guidelines identified through the interpretation of the literature.

**Unit of the analysis:**

Documents (including academic papers, grey literature and case studies)

**N(initial - gathered):**

Not recorded

**N(final - analysed):**

419 documents

**How was the evidence organised:**

For the first purpose, the experts organised and reviewed the information gathered by considering the following questions: i) how is the policy option used in decision-making and implementation? ii) what are the benefits, gaps and challenges of current implementation?, and iii) does the tool help to move beyond the narrow implementations of economic growth? No common database was prepared, but each reviewer kept their own organisation scheme. Information was reviewed and synthesised in multiple tables. For the second purpose, the main topics addressed were: i) how to advance towards the Sustainable Development Goals (through transformative change and diverse values), ii) general values centred action points, iii) gaps and challenges of the operationalization of multiple values for transformative change, iv) multiple options for actions under different types of contexts and v) operationalizing nature’s diverse values in decision making.

**Description of the analysis of the evidence:**

For both purposes, analysis was carried based on expert knowledge, and considering the different documents reviewed, inference of outcomes projected across different sectors and cross sectors on the benefits (and costs of not) incorporating diverse values into decision making was done. Specifically, how diverse values are being perceived within and across sectors, what implications does that have for sectoral and cross-sectoral planning, how inclusive are current policy options of the perspectives of IPLCs and marginalized groups regarding implementation of sectoral/ cross sectoral plans and how might they be more transformative were inferred from the evidence. Furthermore, for the second purpose contextual peculiarities were identified while suggesting guidance on principles and steps that may be followed to operationalize the inclusion of diverse values in decisions related to nature and NCPs.

## **B.27. Review of value related capacity needs and gaps in decision making**

**Table SI.B27:** Summary of review of value related capacity needs and gaps in decision making

| **Specific topics supported:**  Gaps and capacity needs to operationalize the diverse values of nature in decisions | **Type of review:**  Non-structured review fully based on expert criteria |
| --- | --- |
| **DOI of associated data management report:**  10.5281/zenodo.5899737 | **Type of sources:**  Other (chapters 1 - 5 of the values assessment) |
| **Section of the paper where this is covered:**  Section 4 | **Language(s) in which the review was carried:**  English |

**Purpose of gathering the evidence:**

To identify gaps that hinder the operationalization of the diverse values of nature in decisions and capacity development needs to overcome such gaps.

**Process followed to gather the evidence:**

Chapters 1-5 of the IPBES values assessment were reviewed to identify knowledge and operationalization gaps around values and valuation for decision making.

**Process to screen the information gathered:**

Chapters 1-5 were analysed, no filters were applied, all gaps were considered.

**Unit of the analysis:**

Chapters of the IPBES values assessment

**N(initial - gathered):**

5

**N(final - analysed):**

5

**How was the evidence organised:**

A database was created to code and systematise, summarise and organise the gaps. Identified gaps were classified based on the topic they related to, the type of gap it represented, their links to different stages of the decision-making cycle, and the capacities required to address those gaps. Related stakeholders were also identified when possible, although only a limited number of gaps mentioned their relation to specific stakeholders.

**Description of the analysis of the evidence:**

Gaps mentioned in each chapter were identified and consolidated in a spreadsheet. Each gap was analyzed to assess the main topic it represented, the type of gap it represented (operationalization or knowledge gap), its links to the different stages of the decision making cycle (clarify the purpose, recognize diverse values, understand the context, weight up the trade-offs, trace the decision chain, select policy options, find entry points, reflect outcomes), and the capacities required to address those gaps (motivational, analytical, bridging, negotiation, social network and governance). Then, gaps were grouped into clusters (i.e gaps related to: capacities, IPLC, resources, futures, ILK, justice and power, methods, futures, policy instruments, transformation, uptake in policy and values). in accordance with the topic addressed by each of them. Gap clusters were qualitatively described based on the evidence and explained in relation to the capacity dimensions that could help address them.

## **B.28. Review of policy options to leverage multiple values of nature**

**Table SI.B28:** Summary of review of policy options to leverage multiple values of nature

| **Specific topics supported:**  Policy options to leverage diverse values and valuation approaches for transformative governance | **Type of review:**  Combination of reviews:  ● Non-structured review fully based on expert criteria  ● Semi-structured review partially based on expert criteria |
| --- | --- |
| **DOI of associated data management report:**  10.5281/zenodo.4331126 | **Type of sources:**  Peer reviewed literature |
| **Section of the paper where this is covered:**  Section 4 | **Language(s) in which the review was carried:**  English |

**Purpose of gathering the evidence:**

To conceptualise transformative governance and to distil evaluation criteria for the assessment of policy instruments and cross-scale (international) conservation initiatives; and to assess how the incorporation of diverse values in policy instruments can contribute to transformative change.

**Process followed to gather the evidence:**

In the first stage, the main source of evidence used was the core text and the Annexes of Chapter 6 of the IPBES Global Assessment. We reviewed the literature utilised in chapter 5 of the IPBES values assessment[34] regarding transformative governance, as well as the literature from chapter 6 of the IPBES global assessment. This literature was complemented with a search conducted in Google Scholar, using the following search terms: Transformative Environmental Governance OR Transformative Governance and Environmental Values OR Transformative Biodiversity Governance OR Transformative Natural Resource Governance) and with the content of ten sections of chapter 5 of the IPBES values assessment[35]. In the second stage, 37 policy instruments were assessed. The initial list of policy instruments was derived from the IPBES Catalogue of Policy Instruments and Policy Support Tools (IPBES, 2017). Additional policy instruments were added to this list after the screening of the IPBES Global Assessment and regional assessments. Policy instruments were included in the assessment if a direct link to values could be established, and if there was substantial amount of evidence available.

**Process to screen the information gathered:**

Relevant sources were selected based on expert criteria. All the information selected was analysed.

**Unit of the analysis:**

● Academic papers and documents on policy instruments

**N(initial - gathered):**

17 papers and 10 sections of chapter 5 of the IPBES values assessment (Martin et al 2022)

37 policy instruments

**N(final - analysed):**

All pieces of evidence were analyzed

17 papers and 10 sections of chapter 5 of the IPBES values assessment

37 policy instruments

**How was the evidence organised:**

First stage: Reviewed papers were organised in a spreadsheet containing the following fields: source, scope, concept and definition of transformation, proposed criteria to assess transformative potential, other comments. Second stage: Evidence collected from various sources for each policy instrument was organised in one database, containing the following fields: name of instrument, scale of application, source of evidence, body (qualitative summary based on reviewed text), key influencers and actors affected, main approach to valuation, transformative criteria (address status quo, address diverse values, foster institutional change, builds capacities, integrative-adaptive), spread of implementation.

**Description of the analysis of the evidence:**

First stage: Expert judgement was used to create a list of unified criteria which allows to assess policy instruments and cross-scale conservation initiatives in terms of their transformative potential, and to qualitatively describe each of the criteria for transformative governance. Second stage: The potential for incremental or transformational change of policy instruments was evaluated via five criteria identified through a targeted literature review (address status quo, address diverse values, foster institutional change, builds capacities, integrative-adaptive). Each criterion was assessed on a three-point scale: (1) unlikely to meet the criteria if maximum one sub-question could be answered by yes (score=0), (2) medium potential to meet the criteria if 2-3 sub-questions could be answered by yes (score=1), (3) high potential to meet the criteria if three or more sub-questions could be answered. Finally, average scores across the five criteria were calculated. Analysis was done in MS Excel with simple descriptive statistics.

## **B.29. Review of policy instruments to engage with multiple values**

**Table SI.B29:** Summary of review of policy instruments to engage with multiple values

| **Specific topics supported:**  Role of values in transformative governance in the context of policy instruments and other policy initiatives | **Type of review:**  Comprehensive structured review |
| --- | --- |
| **DOI of associated data management report:**  10.5281/zenodo.4331126 | **Type of sources:**  Peer reviewed literature and grey literature |
| **Section of the paper where this is covered:**  Section 6 | **Language(s) in which the review was carried:**  English |

**Purpose of gathering the evidence:**

To assess how cross-scale (international) conservation initiatives can advance the operationalization of diverse values of nature in on-the-ground decisions.

**Process followed to gather the evidence:**

A search was carried out in two stages: First, in Google search, to identify environmental initiatives at global or international scales which: i) oversee or (aim to) influence place-based projects, programmes, policy and decisions related to conservation of biodiversity and ecosystem services; ii) are active over large regional (e.g., continental/subcontinental) or global scales; iii) concern outcomes that link to biodiversity and ecosystem services; iv) advocate knowledge and awareness regarding narrow, plural, or both values within its project activities; and v) have project and institutional documents available in the project domain. To identify initiatives, the following terms were used: “Environmental project”, “Ecosystem service valuation initiative”, “Ecosystem service valuation project”, “Biodiversity project”, “Biodiversity initiative”, ”Nature Project”, “Environmental Project”, “Environmental valuation initiative”, and “Environmental valuation capacity building”. Second, the identified initiatives were looked for in Scopus and Web of Science, using following search string: “[name of initiative]” AND “values” AND “policy” AND “transformative governance” OR “status quo” OR “institutional change” OR “capacity building” OR “integration” OR “adaptation”. When the search yielded no results, or the papers found did not provide sufficient information or evidence, case studies reported on the initiative’s web page were used.

**Process to screen the information gathered:**

Documents that met selection criteria were used.

**Unit of the analysis:**

Case studies

**N(initial - gathered):**

46 case studies

**N(final - analysed):**

43 case studies (comprising 46 initiatives)

**How was the evidence organised:**

Data gathered for the different initiatives was organised into one large database, containing the following fields/questions: Value(s) being addressed; values typology; diverse values present or not, whether or not the vision, mission and “about us” pages considered indigenous and local knowledge; the IPBES region where an initiative was active; dominant decision-making context: Use, Conservation or Development; does it include Targeted Policy Themes?; does it speak to Grand Challenges?; goals/objectives of Initiative; work area boundary (Global, Regional, National, Sub-national , Ecosystem, sector); decision makers targeted.

**Description of the analysis of the evidence:**

First a superficial assessment of 46 cross-scale initiatives was carried out, based on expert judgement, which allowed reviewers to assess how initiatives were generally aspiring to diverse value approaches. Then, to assess how diverse value approaches in policy were used to facilitate transformative governance, specific case studies that documented evidence of policy support for transformative governance were assessed against diverse value approaches and dimensions of transformative governance (i.e., how far they are able to address the status quo, address diverse values, stimulate institutional change, promote capacities, and act in an integrative and adaptive way). This information was used to identify the different ways in which projects incorporated diverse value approaches in policy and practice.

**Endnotes**

[1] This is a review that is based on a clearly planned, structured and consistent (not ad hoc) selection of the evidence. It includes search strings and search terms that define the scope of the review, the different filtering iterations, as well as defined parameters for the selection of the papers to be reviewed. It also includes a pathway to define how the selected literature was reviewed and which were the variables that were considered for the analysis.

[2] This is a review for which most of the steps for the search, selection and analysis of the evidence are outlined, but that rely on expert criteria to guide any one of those stages. They can include literature collected by previous assessment initiatives or that from literature review papers which had their own search and selection criteria.

[3] This is a process in which the review of the literature and its analysis was based on expert knowledge on the topic guided by the content that was being addressed in the values assessment. These reviews did not have a structured approach to defining variables, keywords or search strings at any stage of the review process.

[4] This type of review entails calls for contributions targeted at different types of stakeholders; it includes diverse types of evidence such as scientific literature, grey literature, pieces of art, news articles, declarations, and written contributions. They rely on the evidence that is provided by interested stakeholders or experts summoned to contribute to a specific topic of analysis.

[5] For the full list of search terms see 10.5281/zenodo.4396289

[6] For the full list of codes, see 10.5281/zenodo.4396289.

[7] Kendal, D., & Raymond, C. M. (2019). Understanding pathways to shifting people’s values over time in the context of social-ecological systems. Sustainability Science, 0123456789. https://doi.org/10.1007/s11625-018-0648-0

[8] See 10.5281/zenodo.4071755

[9] See 10.5281/zenodo.4071755

[10] Anderson C.B., Athayde S., Raymond C.M., Vatn A., Arias P., Gould R.K., Kenter J., Muraca B., Sachdeva S., Samakov A., Zent E., Lenzi D., Murali R., Amin A., Cantú-Fernández M. (2022) Chapter 2: Conceptualizing the diverse values of nature and their contributions to people. In: Methodological assessment of the diverse values and valuation of nature of the Intergovernmental Science-Policy Platform on Biodiversity and Ecosystem Services. P. Balvanera, U. Pascual, M. Christie, B. Baptiste, D. González-Jiménez (eds.). IPBES secretariat, Bonn, Germany. XX pages. https://doi.org/10.5281/zenodo.6493134

[11] Michie, S., West, R., Campbell, R., Brown, J., Gainforth, H., 2014. ABC of Behaviour Change Theories Book - An Essential Resource for Researchers, Policy Makers and Practitioners.

[12] Kwon, H.R., Silva, E.A., 2020. Mapping the Landscape of Behavioral Theories: Systematic Literature Review. Journal of Planning Literature 35, 161–179.

[13] For the full list of constructs, see 10.5281/zenodo.4071755.

[14] Lewis-Beck et al. 2004. Conceptualization, Operationalization, and Measurement, in: The SAGE Encyclopedia of Social Science Research Methods. Sage Publications, Inc., 2455 Teller Road, Thousand Oaks California 91320 United States of America. https://doi.org/10.4135/9781412950589.n150

[15] Full list of constructs available at: 10.5281/zenodo.4399907

[16] Anderson C.B., Athayde S., Raymond C.M., Vatn A., Arias P., Gould R.K., Kenter J., Muraca B., Sachdeva S., Samakov A., Zent E., Lenzi D., Murali R., Amin A., Cantú-Fernández M. (2022) Chapter 2: Conceptualizing the diverse values of nature and their contributions to people. In: Methodological assessment of the diverse values and valuation of nature of the Intergovernmental Science-Policy Platform on Biodiversity and Ecosystem Services. P. Balvanera, U. Pascual, M. Christie, B. Baptiste, D. González-Jiménez (eds.). IPBES secretariat, Bonn, Germany. XX pages. https://doi.org/10.5281/zenodo.6493134

[17] Details on the search terms and the web search to generate the database can be found in 10.5281/zenodo.6468906 and 10.5281/zenodo.6468906

[18] See information document 9 focused on valuation methods and approaches

[19] See 10.5281/zenodo.6468906

[20] Intergovernmental Science-Policy Platform on Biodiversity and Ecosystem Services

[21] Rode, J., Gómez-Baggethun, E., & Krause, T. (2015). Motivation crowding by economic incentives in conservation policy: A review of the empirical evidence. Ecological Economics, 109, 80-92. https://doi.org/10.1016/j.ecolecon.2014.11.019

[22] Corte Constitucional de Colombia. (2016). Sentencia T-622/16. https://www.corteconstitucional.gov.co/relatoria/2016/t-622-16.htm

[23] See Balvanera P., Pascual U., Christie M., Baptiste B., Lliso B., Monroy A.S., Guibrunet L., Anderson C.B., Athayde S., Barton D.N., Chaplin-Kramer R., Jacobs S., Kelemen E., Kumar R., Lazos E., Martin A., Mwampamba T.H., Nakangu B., O'Farrell P., Raymond C.M., Subramanian S.M., Termansen M., Van Noordwijk M., Vatn A., Contreras V., González-Jiménez D. (2022) Chapter 1: The role of the values of nature and valuation for addressing the biodiversity crisis and navigating towards more just and sustainable futures. In: Methodological assessment of the diverse values and valuation of nature of the Intergovernmental Science-Policy Platform on Biodiversity and Ecosystem Services. P. Balvanera, U. Pascual, C. Michael, B. Baptiste, D. González-Jiménez (eds.). IPBES secretariat, Bonn, Germany. https://doi.org/10.5281/zenodo.6418971

[24] Bayrak, M. M., & Marafa, L. M. (2016). Ten Years of REDD plus: A Critical Review of the Impact of REDD plus on Forest-Dependent Communities. Sustainability, 8(7), 620. https://doi.org/10.3390/su8070620

[25] Calvet-Mir, L., Corbera, E., Martin, A., Fisher, J., & Gross-Camp, N. (2015). Payments for ecosystem services in the tropics: A closer look at effectiveness and equity. Current Opinion in Environmental Sustainability, 14, 150-162. https://doi.org/10.1016/j.cosust.2015.06.001

[26] Boerner, J., Baylis, K., Corbera, E., Ezzine-de-Blas, D., Honey-Roses, J., Persson, U. M., & Wunder, S. (2017). The effectiveness of payments for environmental services. World Development, 96, 359-374. https://doi.org/10.1016/j.worlddev.2017.03.020

[27] System of Environmental Accounting (Ecosystem Accounting)

[28] Convention on Biological Diversity

[29] See Valuation Atlas in 10.5281/zenodo.6468906

[30] https://seea.un.org/ecosystem-accounting

[31] See Valuation Atlas in 10.5281/zenodo.6468906

[32] The methodological assessment report on scenarios and models of biodiversity and ecosystem services. S. Ferrier, K. N. Ninan, P. Leadley, R. Alkemade, L. A. Acosta, H. R. Akçakaya, L. Brotons, W. W. L. Cheung, V. Christensen, K. A. Harhash, J. Kabubo-Mariara, C. Lundquist, M. Obersteiner, H. M. Pereira, G. Peterson, R. Pichs-Madruga, N. Ravindranath, C. Rondinini and B. A. Wintle (eds.). Secretariat of the Intergovernmental Science-Policy Platform on Biodiversity and Ecosystem Services, Bonn, Germany. 348 pages. https://doi.org/10.5281/zenodo.3235428

[33] IPBES (2019): Global assessment report on biodiversity and ecosystem services of the Intergovernmental Science-Policy Platform on Biodiversity and Ecosystem Services. E. S. Brondizio, J. Settele, S. Díaz, and H. T. Ngo (editors). IPBES secretariat, Bonn, Germany. 1148 pages. https://doi.org/10.5281/zenodo.3831673

[34] Martin, Adrian, O'Farrell, Patrick, Kumar, Ritesh, Eser, Uta, Faith, Daniel P., Gomez-Baggethun, Erik, Harmackova, Zuzana, Horcea-Milcu, Andra-Ioana, Merçon, Juliana, Quaas, Martin, Rode, Julian, Rozzi, Ricardo, Sitas, Nadia, Yoshida, Yuki, Ochieng, Tobias Nyumba, Koessler, Ann-Kathrin, Lutti, Natalia, Mannetti, Lelani, & Arroyo, Gabriela. (2022). Chapter 5. The role of diverse values of nature in visioning and transforming towards just and sustainable futures. Zenodo. https://doi.org/10.5281/zenodo.6633791

[35] Intergovernmental Science-Policy Platform on Biodiversity and Ecosystem Services, IPBES. (2022). Methodological assessment of the diverse values and valuation of nature of the Intergovernmental Science-Policy Platform on Biodiversity and Ecosystem Services. Zenodo. https://doi.org/10.5281/zenodo.6522523

# **Part C. Case study of a multiple values perspective in the restoration and integrated management of Chilika Lagoon, India**

## **C1. Background to the Chilika Lagoon case study**

Chilika, a pear-shaped brackish coastal lagoon in India’s Odisha State. It forms the basis of livelihood security for more than 200,000 fishers and 400,00 farmers living in and around the wetland and its adjoining catchments. Spanning 116,500 ha and flanked by an ephemeral floodplain of 40,000 ha, Chilika is a habitat for at least 1,142 plants and 1,045 animal species, several of which are of high regional and global conservation significance (CDA, 2019). The wetland regularly hosts >1 million wintering migratory birds and is one of the largest congregations of migratory waterbirds in the Central Asian Flyway. It is also one of the two lagoons in the world inhabited by the Irrawaddy dolphin (*Orcaella brevirostris*). The diverse and dynamic assemblage of fish, invertebrate, and crustacean species provide the base of the rich fishery with an average annual yield of 13,000 MT (CDA, 2019). The temple of Kalijai and archaeological sites at Manikpatna, Palur and adjacent areas mark Chilika’s rich maritime heritage. Chilika (along with Keoladeo National Park) was the first Indian wetland to be designated by the Indian government as a Wetland of International Importance under the Ramsar Convention in 1981.

Governance systems in Chilika evolved to manage its diverse and rich fisheries. The traditional fishers developed a system of resource partitioning by setting spatial limits (places to fish), temporal limits (seasonality), gear restrictions (what harvesting gear may be used), and physical limits (what sizes may be fished) (Sekhar 2004). Up to the late 1970s, Chilika’s governance tended to secure the rights of traditional fishers, granting them preferential access to fishing grounds within the lagoon. In 1991, the state government, with the backdrop of pressures for aquaculture development, introduced a policy creating aquaculture areas and thereby legalizing entry of non-traditional fishers into the wetland. Fishing rights in Chilika became contested between the traditional and non-traditional fishers, resulting in a number of violent protests, litigation in courts and ultimately aquaculture being declared illegal with a Supreme Court of India ruling in 1996 that banned aquaculture in any form within the wetland and its 1,000 m periphery (Dujovny, 2009; Samal 2002; Nayak and Berkes, 2011).

Towards the 1970s the lagoon’s connectivity with the Bay of Bengal was progressively impeded, causing a shift to a freshwater dominated state. Channelization of deltaic floodplains, intensification of agriculture and decreasing forest cover in the direct catchments mobilized soil transport and increased the overall sedimentation in the lagoon. The fisheries declined severely, freshwater invasives colonized the Ramsar site, and the traditional resource management systems broke down. In 1993, the Indian government considered placing the Ramsar Site onto the Convention’s Montreux Record, a list of Wetlands of International Importance where changes in ecological character has occurred, is occurring, or is likely to occur.

The rapid decline in ecosystem condition and the associated livelihoods of dependent communities prompted the Odisha state government to constitute the Chilika Development Authority (CDA) as a formal institution mandated to undertake conservation and management of the wetland. Constituted in 1991, the Authority has a mission to “restore and sustainable management of lagoon and its drainage basin based on sound scientific principles through participatory processes” (Kumar et al. 2020).

CDA, working with the state and national governments, a range of scientific organizations, as well as civil society organizations, mobilized the necessary evidence for ecological restoration. Measures put in place since 2000 included opening of a new mouth and dredging of a channel within the northern sector of the lagoon to ensure that riverine sediments are flushed out. These interventions were complemented by a basin scale participatory watershed management programme to contain silt loading from the catchments and enhance resources for community livelihoods (Kumar et al. 2020).

The response of the hydrological intervention and basin management has been rapid and sustained. Based on the positive changes noticed in the ecological character, the national Ministry of Environment, Forest and Climate Change (then the Ministry of Environment and Forests) requested the Ramsar Convention to remove the site from the Montreux Record. Following a Ramsar Advisory Mission in December 2001, the site became the only Asian Ramsar Site, as on date, to be ‘delisted’, being assessed to have sufficiently overcome the factors that had driven it to near social-ecological collapse. Subsequently, these efforts were also recognized with the Ramsar Wetland Conservation Award and the Evian Special Prize for ‘wetland conservation and management initiatives’.

After initial trophic bursts, the annual fish landing stabilized at nearly 13,000 MT (CDA, 2019). Annual CDA censuses of Irrawaddy dolphins within Chilika reported an increase from 89 to 151 individuals between 2003 and 2022, as well as increases in habitat use, improvements in breeding and dispersal, and declines in mortality rates (CDA monitoring records, unpublished). Sea grass meadows expanded from 20 km2 in 2000 to 172 km2 by 2022, along with a significant decline in freshwater invasive species (CDA monitoring records, unpublished).

In the post-restoration phase, the management of this Ramsar Site is guided by an integrated management plan. In 2012, the Chief Minister of Odisha (also the Chairman of CDA) launched this initiative. Through dedicated capacity building, conflict resolution, and trust building, CDA enabled formulation of watershed management plans and also provided resources for their implementation. An intensive awareness campaign on the multiple values and functions of the wetland system, particularly amongst the fishers and school children, was undertaken in participation with civil society. A visitor centre was constructed as a hub for these activities. Specific initiatives for managing tourism by building capacity of the boatmen association were also undertaken. Additionally, a code of conduct for dolphin watching was developed, and CDA has also strengthened fishing infrastructure through the construction of landing centres and jetties. To support systematic management, an intensive hydrological and ecological monitoring programme has been put in place, coordinated through the Wetland Research and Training Center built on the shorelines of Chilika in 2002. Over the years, CDA has also established collaborations with >50 organizations of international and national repute to support scientific studies related to various dimensions (Kumar et al 2020).

## **C2. Identifying the multiple values associated with Chilika wetland restoration with the Values Typology**

Chilika management from the government’s perspective was traditionally structured around an anthropocentric worldview emphasizing *living from* the wetland as an economic asset for food and employment. The traditional fisher communities, however, also emphasise *living with* the wetland, and express relational values through fishing and traditional management, in connection to their traditional ecological knowledge about fish species’ behaviour. Yet, the government continued to favour a market-oriented instrumental value perspective, introducing new forms of fisheries (e.g., aquaculture), which over time led to marginalization of traditional fishers’ worldviews and values about the wetland. The CDA, in the post-ecological restoration phase, manages the Ramsar Site on the basis of a range of values, including anthropocentric, ecocentric, and pluricentric worldviews that are also reflected by a range of broad and specific values (as summarized in Figure SI.C1 and Table SI.C1).


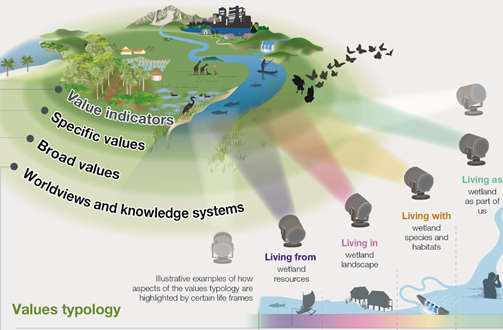


**Figure SI.C1:** The typology of values associated with the Chilika Lagoon, India.

Table SI.C1. Examples of the different value layers and value types associated with Chilika Lagoon for different stakeholder groups holding diverse worldviews.

| **Worldviews** | **Ways through which people conceive and interact with the world** | **Living from**  **Chilika (resources)**    **(Anthropocentric)** | **Living in**  **Chilika (landscape)**    **(Anthropocentric)** | **Living with**  **Chilika (species and habitats)**    **(Bio/ ecocentric)** | **Living as**  **Chilika (as a part of us)**    **(Pluricentric)** |
| --- | --- | --- | --- | --- | --- |
| Knowledge systems | Bodies of knowledge, practices and beliefs    (academic, indigenous, local) | Scientific knowledge of Chilika fisheries, aquatic vegetation    Traditional and indigenous knowledge on fishing grounds, fishing techniques | Scientific knowledge of landscape features of Chiliks and its surroundings    Traditional knowledge on floods and silt distribution in Mahanadi floodplains and interactions with Chilika | Scientific knowledge of species and habitat    Traditional knowledge of species interactions underlying co-existence of Chilika fishers with birds and Irrawaddy Dolphins | Chilika as a part of poetry and folklore    Sacredness of Chilika |
| Broad values  guiding Chilika restoration and management | Guiding principles | Maintaining a non-declining harvest of wetland resources    Fair value realization to the fishers in the market chain    Preventing detrimental resource use practices | Ensuring connectivity between the marine environment (Bay of Bengal) and riverine environment (Mahanadi floodplains)    Aligning land use of Chilika catchment with Chilika ecosystem functioning | Enhancing species diversity | Protecting cultural identities and relational linkages with Chilika |
| Specific values guiding Chilika restoration and management | Judgements regarding the importance of nature in particular situations    Instrumental (means to an end, nature as a resource / asset, satisfaction of needs and preferences, usefulness for people)    Intrinsic (agency of other than humans, inherent worth of biodiversity as ends in-and of-themselves)    Relational (importance of desirable, meaningful, and other reciprocal relationships) | Fin-fish and shell-fish landing and their monetary value    Wetland tourism, adherence to established code of conduct, contribution to local economy    Extent of wetland area under illegal fishing practices | Salinity gradient    Silt from inflowing catchments | Population and habitat use by Irrawaddy dolphins, waterbirds    Fish migration pathways    Area under key habitats | Maintenance of sacred places, and those of heritage value |
| Value indicators | Quantitative measures and qualitative descriptors    (Biophysical, Monetary, Sociocultural) | Tonnes of fin-fish and shellfish landed in a year, monetary value of landings, and accrual to the fisher cooperatives    Total tourist inflow, revenue generation, and accrual to the local economy    Number of people using Chilika to commute within islands    Instances of violation of code of conduct by boat operators and tourists    Wetland area under illegal aquaculture | Wetlands research center continues to monitor wetland ecosystem health and generates data on various wetland features  Number of mouths and their width    Salinity values in different parts of the wetland    Tidal flux around the Chilika mouth    Interannual water level variation    Quantity of silt received into the wetland | Species richness    Area under sea-grass meadows    Area under mangroves    Waterbird populations    Irrawaddy dolphin counts | Communities paying obeisance at Kalijai temple |

## **C3. The Chilika Wetland restoration policy cycle**

Ecological restoration and subsequent management of the Chilika Lagoon have involved different stages of the policy cycle with the CDA playing the crucial role of agenda setting, creating mechanisms for consideration of diverse worldviews and values in decision-making processes, and creating mechanisms for review and adaptation (Table B2).

Table SI.C2. Mapping of the policy cycle with elements of Chilika Lagoon restoration and management decisions

| **Policy cycle element** | **Key elements for Chilika restoration and management** | **Role of valuation** |
| --- | --- | --- |
| Agenda setting | Until the 1990s, management of the Chilika Lagoon was centered around fisheries. It was designated as a Ramsar Site in 1981, but placed under Montreux Record in 1993 due to deteriorating lagoon conditions. In 1991, the Odisha government constituted Chilika Development Authority (CDA) for ecological restoration and holistic management of the wetland ecosystem. | **Valuation to inform**  CDA recognized that management of instrumental values (fisheries) could not be done without assessing intrinsic values (related to (biophysical indicators of Chilika’s ecological character) and taking into account the values perspectives of diverse stakeholders held, including instrumental (e.g., fish catch and market value) and relational values (e.g., cultural identity, sacredness). |
| Policy formulation | CDA implemented an ecological restoration plan in 2000 to improve connectivity between the lagoon and Bay of Bengal. Post restoration, CDA put in place a monitoring system to generate long term information on ecological and hydrological features. A range of specific ecological and socioeconomic assessments were commissioned to provide insights into ecosystem dynamics and stakeholder interactions in the wetland. | **Valuation to design**  The design of integrated management plan included actions components addressing: 1) Intrinsic values (e.g., through components focused on habitat conservation, water management, and conservation of watersheds); 2) instrumental values (e.g., through components on sustainable fisheries development and ecotourism development), and 3) relational values (e.g., through components on cultural-heritage values conservation, and communications and outreach) |
| Policy adoption | In 2012, the CDA adopted an integrated management plan to guide actions for conservation and wise use. | **Valuation to decide**  The integrated management plan provided a mechanism for considering multiple value perspectives (e.g., those of primary stakeholders, politicians, government departments, and civil society), while deciding interventions for the wetland’s wise use. |
| Policy implementation | Integrated management plan has been in implementation since 2012 with resources from the state and central government. Various components are implemented by respective line departments with the CDA playing a coordination role, and implementing research and monitoring and evaluation plan. | **Valuation to inform, design and decide**  The implementation of management plan components, though aligned with individual sector outcomes, is assessed by the governing body of the CDA. This mechanism allows for considering value interactions, and resolving value conflicts during the course of implementation. CDA also serves to communicate the multiple values of Chilika in sector plans (e.g., tourism development, water resources development, disaster risk reduction). |
| Policy evaluation | Implementation of the integrated management plan includes monitoring and evaluation, which forms the basis of evaluating management effectiveness. CDA produces a Report Card every two years which summarizes the monitoring data into health scores. | **Valuation to inform**  The effectiveness of wetland management is assessed by reporting on a range of values and value indicators including: 1) intrinsic values (e.g., species richness, extent of key habitats,habitat use by target species), 2) instrumental values (e.g., shell and fin fish catch, wetland tourism), and 3) relational values (e.g., quality of sacred spaces, conflicts avoided). |

## **C4. Leverage points for the transformation of the wetland social-ecological system towards sustainability**

Integrated management of Chilika is aimed at ‘wise use’, defined in the Ramsar Convention text as “maintenance of ecological character, achieved through implementation of ecosystem approaches, within the context of sustainable development” (Ramsar, 2005). Recognizing diverse values (leverage point 1) and integrating them into decisions regarding management of the Ramsar site (leverage point 2) is made is central to achieving this goal. The formation of the CDA as a new institution (leverage point 3) has allowed to mobilize the first leverage point by recognizing that Chilika is not just a resource providing food and employment to those living in and around it (instrumental values), but also a complex social-ecological system providing habitats to diverse lifeforms (intrinsic values) , a source of individual and community identity for fishers and cultural heritage (relational values). Implementation of integrated management plan post-ecological restoration of 2000 has sought to address the diverse values by including specific actions for conserving species and habitats, sustainable development of fisheries, capacity development of traditional fishers, nature-based tourism, conserving wetlands catchments, and communication and outreach on diverse wetland features. The decision-making within the Authority is steered by its Governing Body, headed by the Chief Minister with elected representatives, secretaries of concerned government departments, administrators, experts, and representatives of fisher cooperatives as members. This allows different values being discussed and negotiated towards the wetland’s management. Despite being a state agency, the establishment of the CDA can be seen as an enabling institution, convening stakeholders around wetland management, building capacity and outreach and providing the science and knowledge base for making informed decisions. For example, recently the Authority demonstrated application of its regulatory functions to demolish nearly 120 km2 of illegal prawn enclosures within the lagoon. However, still much remains to be done in terms of shifting social norms and goals linked with wetlands management (leverage point 4). The traditional norms for wetlands management held by fishers, which were also ecologically aligned, have gradually eroded, and the newer arrangements (majorly influenced by significant tourism growth) are once again risking the perpetuation of the excessive predominance of market-based instrumental values viz-a-viz other important values about the wetland.

**References**

CDA (2019). *Chilika - Integrated Management Plan (2019-2024)*. Chilika Development Authority, Bhubaneshwar, Odisha

Dujovny, E. (2009). *The Deepest Cut: Political Ecology in the Dredging of a New Sea Mouth in Chilika Lake, Orissa, India.* Conservation and Society 7 (3): 192. doi:10.4103/0972-4923.64736.

Kumar, R., Finlayson, C.M., Pattnaik, A.K. (2020). *Ecological Characterization of Chilika: Defining Strategies and Management Needs for Wise Use.* In: Finlayson, C., Rastogi, G., Mishra, D., Pattnaik, A. (eds) Ecology, Conservation, and Restoration of Chilika Lagoon, India. Wetlands: Ecology, Conservation and Management, vol 6. Springer, Cham. <https://doi.org/10.1007/978-3-030-33424-6_3>

Nayak, P. K., and Berkes, F.. (2011). *Commonisation and Decommonisation: Understanding the Processes of Change in the Chilika Lagoon, India.* Conservation and Society 9 (2): 132. doi:10.4103/0972-4923.83723.

Ramsar. 2005. Resolution IX . 1 Annex A: *A Conceptual Framework for the wise use of wetlands and the maintenance of their ecological character.* The Ninth Meeting of Conference of Parties. Kampala, Uganda: The Ramsar Convention on Wetlands.

Samal, K.C. (2002). *Shrimp Culture in Chilika Lake: Case of Occupational Displacement of Fishermen.* Economic and Political Weekly 37 (18): 1714–18. http://www.jstor.org/stable/4412064.
